# Selective Modulation of Fear Memory in Non‐Rapid Eye Movement Sleep

**DOI:** 10.1002/advs.202400662

**Published:** 2024-10-09

**Authors:** Qiyu Zheng, Yuhua Huang, Changrui Mu, Xiaoqing Hu, Cora Sau Wan Lai

**Affiliations:** ^1^ School of Biomedical Sciences LKS Faculty of Medicine The University of Hong Kong Hong Kong SAR China; ^2^ Advanced Biomedical Instrumentation Centre Hong Kong Science Park Shatin, New Territories Hong Kong China; ^3^ Department of Psychology Faculty of Social Sciences The University of Hong Kong Hong Kong SAR China; ^4^ The State Key Laboratory of Brain and Cognitive Sciences The University of Hong Kong Hong Kong SAR China

**Keywords:** fear, sleep, slow oscillation, spindle, targeted memory reactivation

## Abstract

Sleep stabilizes memories for their consolidation, but how to modify specific fear memory during sleep remains unclear. Here, it is reported that using targeted memory reactivation (TMR) to reactivate prior fear learning experience in non‐slow wave sleep (NS) inhibits fear memory consolidation, while TMR during slow wave sleep (SWS) enhances fear memory in mice. Replaying conditioned stimulus (CS) during sleep affects sleep spindle occurrence, leading to the reduction or enhancement of slow oscillation‐spindle (SO‐spindle) coupling in NS and SWS, respectively. Optogenetic inhibition of pyramidal neurons in the frontal association cortex (FrA) during TMR abolishes the behavioral effects of NS‐TMR and SWS‐TMR by modulating SO‐spindle coupling. Notably, calcium imaging of the L2/3 pyramidal neurons in the FrA shows that CS during SWS selectively enhances the activity of neurons previously activated during fear conditioning (FC+ neurons), which significantly correlates with CS‐elicited spindle power spectrum density. Intriguingly, these TMR‐induced calcium activity changes of FC+ neurons further correlate with mice freezing behavior, suggesting their contributions to the consolidation of fear memories. The findings indicate that TMR can selectively weaken or strengthen fear memory, in correlation with modulating SO‐spindle coupling and the reactivation of FC+ neurons during substages of non‐rapid eye movement (NREM) sleep.

## Graphical abstract

Targeted memory reactivation via replaying of learning‐related sensory cue in NREM sleep substages induces opposite effects on fear memory consolidation. This process is associated with the changes of synaptic plasticity and sleep spindles in the frontal cortex. Furthermore, the involvement of pyramidal neurons in the frontal cortex is crucial for the effects, and the reactivation of learning associated neurons induced by targeted memory reactivation correlates with animal fear response.

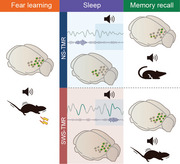

## 1 Introduction

Sleep contributes to learning and memory consolidation. During sleep, neurons involved in wakeful experiences are reactivated in various cortical and subcortical brain regions.^[^ ^1^, ^2^, ^3^, ^4^, ^5^ ^]^ Neuronal reactivation, together with the rhythmic oscillatory patterns during sleep, e.g., slow oscillation (SO), sleep spindle, and sharp‐wave ripple, are key mechanisms in promoting memory consolidation and information integration.^[^ ^6^, ^7^, ^8^, ^9^, ^10^ ^]^ Intriguingly, sleep also provides a unique time window to modify specific memory using a technique called targeted memory reactivation (TMR), in which wakeful learning‐related sensory cues are replayed to the sleeping brain to reactivate their associated memories.^[^ ^11^, ^12^, ^13^ ^]^ In human, TMR in post‐learning non‐rapid eye movement (NREM) sleep promotes memory consolidation via SO‐spindle couplings in tasks including spatial navigation, motor sequence learning, and declarative memories.^[^ ^14^, ^15^, ^16^, ^17^ ^]^

However, opposite TMR effects in NREM sleep have been found in fear or negative emotional memory in both humans and rodents. In human studies, re‐exposure to odor cues associated with prior fear learning weakened fear memory when the cues were applied during slow wave sleep (SWS, or N3 substage of NREM).^[^ ^18^, ^19^ ^]^ In contrast, another study showed that SWS‐TMR improved the performance of negative memories.^[^ ^20^ ^]^ Similarly, rodent research also revealed contrasting outcomes. Mice that underwent associative learning between foot shock and conditioned stimulus (e.g., auditory tone) expressed a significantly reduced response to the conditioned stimulus (i.e., decreased freezing time) after TMR in immediate post‐learning NREM sleep.^[^ ^21^ ^]^ In another study, NREM‐TMR 24 h after fear conditioning instead reinforced fear memory in mice.^[^ ^22^ ^]^ While these contrasting findings could potentially be attributed to the different time window of TMR administration (immediate versus 24 h post‐learning sleep), whether TMR during different NREM substages (non‐slow wave sleep (NS) or SWS) may impact the fear memory has not been investigated. Interestingly, another study further demonstrated that selective TMR during post‐learning SWS in rats significantly increased the freezing time after fear conditioning,^[^ ^23^ ^]^ further highlighting the importance of considering different NREM substages.

Moreover, beyond the behavioral expressions, how TMR affects synaptic plasticity and neuronal activity, and how such activity influences memory consolidation remain elusive. The different NREM sleep substages are characterized by distinct neural activity patterns, such as varying proportions and dynamics of neural oscillations, that is critical for memory consolidation.^[^ ^24^, ^25^, ^26^ ^]^ Therefore, here we used the classic auditory‐cued fear conditioning to investigate the effects of TMR in different substages of NREM sleep on animal behavior, neural oscillations, and specific neuron responses in mice.

## 2 Results

### 2.1 Classification of NREM Substages in Mice

**Figure 1:**
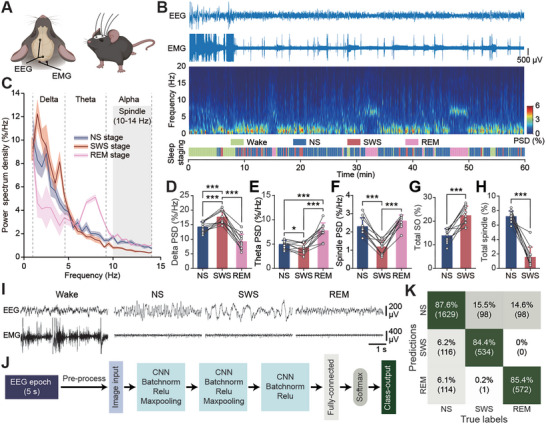
Classification of sleep substages. A) Schematic of the locations for EEG/EMG electrodes implantation on mice. B) Example of EEG/ EMG traces (upper), EEG power spectrogram (middle) and staging of brain states (bottom) during 1‐hour post‐fear learning sleep period. C) Normalized EEG power spectrum density in delta band (0.8 – 4 Hz), theta band (4 – 8 Hz), and alpha band (8 – 14 Hz) of different sleep stages in white noise‐only (WN) group. The oblique line area shows spindle band (10 – 14 Hz). Data shown as mean ± SEM. D–F): Quantifications of power spectrum density of delta, theta, and spindle bands in WN group. G,H) Quantifications of time of oscillation subtypes in NS or SWS over the time of NS or SWS in WN group. For (D) to (H), data shown as mean ± SD; number of each group = 11; ***P < 0.001; data sets with normality use two‐tailed paired *t*‐test; without normality use Wilcoxon matched‐pairs signed rank test. I) Examples of EEG/EMG traces for sleep staging: Wake, NS, SWS, and REM sleep. J) Architecture of CNN deep learning model for sleep substages classification. K) Confusion matrix for the trained CNN model on held‐out testing data. The number of epochs is shown in parentheses. The overall accuracy was around 86.5%.

Different from the standard human sleep staging where NREM sleep can be sub‐divided into N1, N2, and SWS, the distinction of NREM substages was less clear in most rodent research.^[^ ^27^ ^]^ Considering the enhanced fear memory after selective SWS‐TMR in rats,^[^ ^23^ ^]^ here we divided NREM sleep into SWS and non‐SWS (NS) based on mice electroencephalography (EEG) features (see methods; **Figure** **1**A,B,I). The electrophysiological data was continuously monitored and scored real‐time (online) for the targeted sleep substages, and the data was further visually labelled and analyzed retrospectively (offline) (Figure 1B‐H). Specifically, after the Wake stage was ruled out based on prominent electromyography (EMG) activities, the rapid eye movement (REM) sleep was characterized by the low‐amplitude, high‐frequency theta EEG activity, while the remaining epochs were identified as NREM sleep. SWS was identified if there were at least two continuous, synchronized, high‐amplitude slow waves occurred within a 5‐second epoch, and accordingly, the remaining NREM periods were classified as NS (Figure 1B,I). Our offline analysis showed that, consistent with human studies,^[^ ^28^, ^29^ ^]^ SWS in mice exhibited significantly higher power spectrum density (PSD) in delta band (0.8‐4 Hz) and longer duration of SO than the NS (Figure 1D,G); while NS showed significantly longer duration of spindles than SWS (Figure 1H). Sleep cycles in rodents are shorter and more fragmented than in humans.^[^ ^4^ ^]^ We therefore examined the duration of continuous NS and SWS epochs and found that the duration of continuous NS epochs in mice was significantly longer than SWS (Figure S1B, Supporting Information). The clustering analysis of SWS epochs further revealed that more than 78% of continuous SWS epochs were longer than 10 s (Figure S1A, Supporting Information), suggesting the feature of temporally clustering of SWS in mice. We next validated the reliability of our visual labelling by employing a convolutional neural network (CNN) deep learning approach. We incorporated the graphical representations of manually scored sleep EEG epochs as the inputs for the CNN model (Figure 1J), the architecture of which was based on previously designed layers for sleep staging.^[^ ^30^ ^]^ Our results indicated that the trained deep learning model effectively captured the features of distinct sleep substages, obtaining an overall accuracy of ≈86.5% for the testing data (Figure 1K), suggesting that the classification of sleep substages is both rational and achievable. In addition, the online sleep stage scoring showed ≈90% accuracy benchmarked to offline staging analysis (Figure S1C, Supporting Information), demonstrating the accuracy of NREM sub‐staging.

### 2.2 TMR in SWS Strengthens, while TMR in NS Weakens Fear Memory

**Figure 2:**
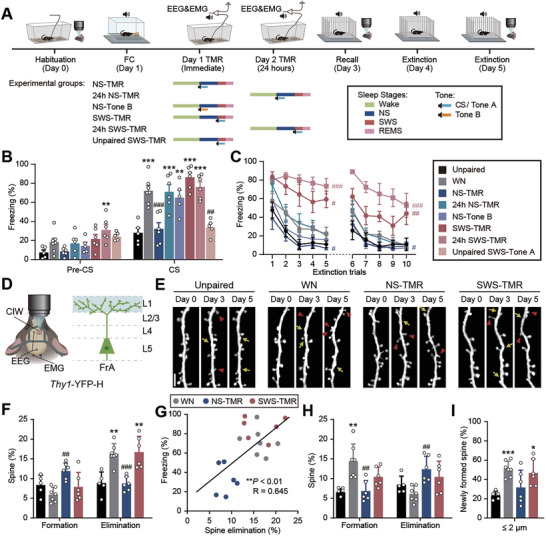
TMR in NS and SWS shows opposite effects on fear learning. A) Upper: Timeline showing the behavioral training and imaging sessions. Bottom: TMR paradigms in Day 1 or Day 2 for different experimental groups. CS/Tone A is the tone used in paired or unpaired fear conditioning; Tone B is a novel tone that mice is not exposed before. B) Freezing response (percentage of time) in the recall test during the pre‐CS and CS at Day 3. C) Freezing response in the extinction trials during CS at Day 4 and 5; two‐way ANOVA was used for comparison over 5 trials or 10 trials. D) Left: Schematic of the locations for EEG/EMG electrodes and cranial imaging window implantation for in vivo two‐photon imaging on the frontal association cortex (FrA). Right: Schematic of the imaging depth (cyan) of the apical dendrites of layer (L) 5 pyramidal neurons expressing YFP. E) Representative images of apical dendrites of L5 pyramidal neurons in the FrA. Asterisks denote filopodia (excluded in spine analysis). Yellow arrows denote spine formation and red arrowheads denote spine elimination, compared to the previous timepoint. Scale bar, 5 µm. F) Rates of dendritic spine formation and elimination induced by fear conditioning at Day 3. G) Correlation between rates of spine elimination induced by fear conditioning and freezing in recall test at Day 3; linear regression was used to plot the line and two‐tailed Pearson's correlation test was used for analysis. H) Rates of dendritic spine formation and elimination after extinction at Day 5. I) Proportion of newly formed spines induced by fear extinction located within 2 µm from the closest sites of spine elimination induced by fear conditioning. Data shown as mean ± SEM for (B) and (C), mean ± SD for (F), (H), and (I). Unpaired, *n* = 5, WN, *n* = 7, NS‐TMR, *n* = 5, 24 h NS‐TMR, *n* = 5, NS‐Tone B, *n* = 5, SWS‐TMR, *n* = 6, 24 h SWS‐TMR, *n* = 5, Unpaired SWS‐Tone A, *n* = 5; *P < 0.05, **P < 0.01, ***P < 0.001, marked groups compared to Unpaired group (B, F, H, and I); ^#^ *P* < 0.05, ^##^ *P* < 0.01, ^###^ *P* < 0.001, marked groups compared to WN (B, C, F, and H); data sets with normality and homogeneity of variance use one‐way ANOVA, Tukey's multiple comparisons *post hoc* test; with normality and without homogeneity of variance use Welch's ANOVA, Dunnett's T3 multiple comparisons test.

The frontal association cortex (FrA) has been shown to be involved in fear learning.^[^ ^31^, ^32^, ^33^, ^34^ ^]^ Previous study showed that dendritic spine plasticity in the FrA is correlated with fear learning and freezing behavior.^[^ ^34^ ^]^ FrA receives direct inputs from basolateral amygdala (BLA) and sensory cortices and it also have direct projection to subcortical areas, including thalamic reticular nucleus (TRN) (Figure S2, Supporting Information), where sleep spindle is generated.^[^ ^31^, ^35^, ^36^, ^37^ ^]^ Therefore, we next evaluated the effects of TMR in distinct NREM sleep substages, namely NS and SWS, on fear memory consolidation and dendritic spine plasticity in the FrA based on its role in fear learning and optical accessibility by two‐photon imaging. *Thy1*‐YFP H line mice with the expression of yellow fluorescent protein (YFP) in layer (L) 5 pyramidal neurons in the neocortex were implanted with EEG/EMG electrodes and cranial window in the FrA for sleep staging and for two‐photon imaging of dendritic spines (**Figure** **2**A,D). After auditory‐cued fear conditioning (FC), mice were placed in the pre‐habituated cage for 4‐hour EEG/EMG recording, during which we presented the conditioned stimulus (CS, an auditory cue) in SWS or NS with background white noise throughout the 4‐hour recording period (Figure 2A). A white noise‐only (WN) group served as a control group. The CS was played and lasted 5–30 s based on the duration of each SWS and NS epoch (see methods). We controlled the time of TMR in each group so that the total number and duration of CS applied were not significantly different between the SWS‐TMR and NS‐TMR groups (Figure S1D,E, Supporting Information), even though the duration of discrete sleep epoch during SWS and NS were significantly different (Figure S1F, Supporting Information). There is no significant difference in the total sleep time during the 4‐hour recording among WN control group and TMR groups (Figure S1G, Supporting Information). After TMR, mice were returned to home cage and left undisturbed. Fear recall tests were conducted 48 hours after FC (Day 3). In Day 4 & 5, mice were subjected to fear extinction. Two‐photon imaging was performed on Day 0, Day 3 after the recall test and Day 5 after the last trial of fear extinction, respectively (Figure 2A).

We found that the TMR during NS significantly reduced freezing responses during fear recall test when compared to WN control group (32.2 ± 6.4% in NS‐TMR versus 72.1 ± 5.4% in WN, *P* < 0.001, Figure 2B). Interestingly, there was no significant difference in freezing responses if a learning‐irrelevant tone (Tone B) was presented during NS in the first 4‐hour post‐learning sleep (NS‐Tone B), or when the CS was presented during the NS in the next day (24 h NS‐TMR) (Figure 2A). These results suggest that the impairment of fear memory consolidation in NS‐TMR is CS/cue‐specific and is time‐dependent within the 24‐hour post‐learning time window. TMR in REM sleep also showed no effects on mouse fear memory behavior (Figure S3, Supporting Information). In contrast, mice that received SWS‐TMR slightly increased the freezing time during the Day 3 recall test, though this increase did not reach statistical significance (86.2 ± 4.8% in SWS‐TMR versus 72.1 ± 5.4% in WN, *P* = 0.61, Figure 2B). Notably, SWS‐TMR significantly increased freezing responses during Day 4 & 5 fear extinction trials than the WN group (two‐way ANOVA, *F* (1, 11) = 8.792, *P* < 0.05 for Day 4, *F* (1, 11) = 10.55, *P* < 0.01 for Day 4 and 5, Figure 2C). Thus, mice in the SWS‐TMR group showed a higher resistance toward fear extinction training, suggesting that SWS‐TMR enhances fear memory consolidation. Memory replays occur during post‐learning sleep;^[^ ^1^, ^38^ ^]^ hence we asked if the memory enhancement effect of SWS‐TMR can be ascribed to the reinforced association of fear memory replay and CS presentation while asleep. To answer this, we presented the same tone to an Unpaired group during SWS (Unpaired SWS‐Tone A: mice received tone A and foot‐shocks but without association during unpaired training, Figure 2A). Our data showed that the presentation of unpaired Tone A during SWS did not enhance fear response during fear recall (33.9 ± 3.8% in Unpaired SWS‐Tone A versus 28 ± 5.1% in Unpaired, *P* = 0.99, Figure 2B), indicating that SWS‐TMR strengthens memory by initiating cue‐associated memory reactivation,^[^ ^39^ ^]^ rather than through reinforcing novel associations during sleep. Surprisingly, SWS‐TMR at 24 h after fear conditioning (24 h SWS‐TMR) showed a stronger resistance to fear extinction than the WN (two‐way ANOVA, *F* (1, 11) = 40.33, *P* < 0.001, Figure 2C), suggesting that the memory enhancement effect of SWS‐TMR is beyond 24 h post‐fear learning.

Learning‐induced plasticity of individual synapses on dendritic branches are crucial for memory storage.^[^ ^40^ ^]^ A prior study showed that FC and fear extinction elicit an opposite dendritic spine plasticity in the FrA: FC induced significantly more dendritic spine elimination, whereas fear extinction induced more spine formation within a 2 µm proximity from the FC‐induced spine elimination location on dendritic branches, demonstrating a location‐ and cue‐specific dendritic spine plasticity.^[^ ^34^, ^40^ ^]^ To understand the opposite effects of TMR in the first 4 hours post‐learning NS and SWS, we examined the dendritic spine plasticity in the FrA after FC and extinction (Figure 2A,D,E). We found that NS‐TMR significantly lowered FC‐induced dendritic spine elimination (8.7 ± 1.9% in NS‐TMR versus 16.3 ± 2.6% in WN, *P* < 0.001 Figure 2F); while after extinction, SWS‐TMR reduced the amount of extinction‐induced dendritic spine formation that showed no significant difference when compared to Unpaired control group (WN versus Unpaired, *P* < 0.01; SWS‐TMR versus Unpaired, *P* = 0.19, Figure 2H). Furthermore, the increase of location‐specific spine formation was also reduced in NS‐TMR group (WN versus Unpaired, *P* < 0.001; NS‐TMR versus Unpaired, *P* = 0.80, Figure 2I). Although SWS‐TMR failed to form new spines as many as WN control induced by extinction (Figure 2H), the location‐specificity was still maintained, as ≈50% of new spines were within 2 µm proximity to FC‐induced eliminated spines, similar to WN (Figure 2I). Considering that extinction induced dendritic spine formation in a cue‐ and location‐ specific manner,^[^ ^34^ ^]^ these data suggest that the newly formed spines in SWS‐TMR are location‐specific and fear learning‐related. In addition, the rate of FC‐induced spine elimination was significantly correlated with freezing response (Figure 2G); while the rate of fear extinction‐induced spine formation was also negatively correlated with the change of freezing responses after one day's extinction training (Figure S4, Supporting Information). These data show that the opposite effects of NS‐TMR and SWS‐TMR in fear memory consolidation not only reflect in freezing behavior but also in dendritic spine plasticity in the FrA.

### 2.3 NS‐TMR Reduces, while SWS‐TMR Enhances SO‐Spindle Couplings During Sleep

**Figure 3:**
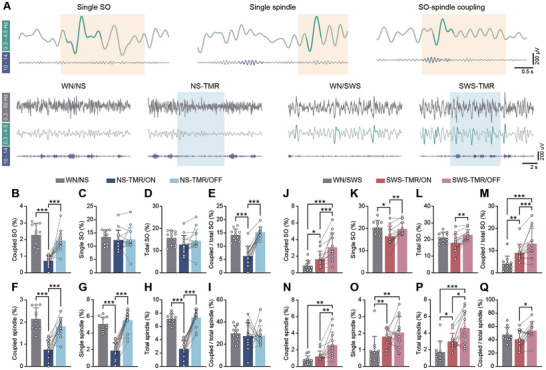
The effects of NS‐TMR and SWS‐TMR on oscillatory patterns. A) Upper: examples of EEG traces of single SO, single spindle, and SO‐spindle coupling in distinct filtering bands. The darkened color denotes identified SO or spindle. Beige shadings show the coupling time window of SO, which covers the pre‐0.5 s to the post‐1.5 s of the SO peaks. Bottom: Representative EEG traces in each group, filtered by distinct bands. The light blue shadings show the TMR period. B–D) and F–H): Quantification of time of oscillation subtypes over the time of NS. (E) and I): Proportion of the time of coupled SO or coupled spindle over total SO or spindle. J–L) and N–P): Quantification of time of oscillation subtypes over the time of SWS. M) and Q): Proportion of the time of coupled SO or coupled spindle over total SO or total spindle. For (B) to (Q): Data shown as mean ± SD. WN/NS, *n* = 11, NS‐TMR/ON, *n* = 14, NS‐TMR/OFF, *n* = 14, WN/SWS, *n* = 11, SWS‐TMR/ON, *n* = 12, SWS‐TMR/OFF, *n* = 12. *P < 0.05, **P < 0.01, ***P < 0.001; when compared data between ON and OFF labeled groups: data sets with normality use two‐tailed paired *t*‐test; without normality use Wilcoxon matched‐pairs signed rank test; when compared to WN/NS or WN/SWS, data sets with normality and homogeneity of variance use two‐tailed unpaired *t*‐test; with normality and without homogeneity of variance use unpaired *t*‐test with Welch's correction; without normality use Mann Whitney test.

As memory reactivation and consolidation were often associated with SO, spindle, and SO‐spindle coupling events,^[^ ^41^, ^42^, ^43^, ^44^ ^]^ we next examined how these neural oscillations were associated with the opposite effects of NS‐ and SWS‐TMR. We extracted the SO, spindle, and SO‐spindle coupling events as previously described^[^ ^7^, ^45^ ^]^ (see methods; and **Figure** **3**A) and measured the percentage of time dedicated to SO and spindle over the respective sleep stages based on offline analysis. To gain deeper insight into the relationship between TMR and sleep rhythms, with an emphasis on rhythm coupling events, we further characterized coupled and uncoupled (single) SOs/spindles events, considering if an event was coupled with another in a specific time window (Figure 3A). The percentage of time of these events in WN group were then compared to the CS presentation period (ON) and non‐CS presentation period (OFF) during NS‐TMR and SWS‐TMR, respectively.

Our data showed that the time of coupled SO and spindles, including single, coupled, and total spindle counterparts, significantly declined during TMR ON period in NS‐TMR (*P* < 0.001, Figure 3B,F‐H). In contrast, the total and single SOs remained relatively unaffected compared to the WN group in NS. (Figure 3C,D). Moreover, the ratio of coupled SOs instead of coupled spindles was reduced when TMR was presented in NS (Figure 3E,I), suggesting that NS‐TMR diminishes SO‐spindle couplings by inhibiting spindle generation. Additionally, our data revealed that the changes in oscillatory events returned to levels comparable to WN when TMR was paused (NS‐TMR/OFF, Figure 3B‐I), highlighting the transient nature of NS‐TMR's impact.

On the contrary, SWS‐TMR triggered significantly more SO‐spindle couplings (Figure 3J,N). We observed that, when TMR was presented during SWS, the duration of total spindle increased (Figure 3P). Furthermore, the ratio of coupled SO rose (Figure 3M) without significant changes of the time of total SO (Figure 3L), suggesting that SWS‐TMR promotes spindle generation, leading to a greater percentage of SOs coupling with spindles. Intriguingly, unlike the transient impact of NS‐TMR on neural oscillations, the elevated levels of spindles and SO‐spindle couplings persisted or even further intensified during SWS‐TMR/OFF period (i.e., 1.63 ± 0.98% of coupled SO in SWS‐TMR/ON versus 3.07 ± 1.74% of coupled SO in SWS‐TMR/OFF, *P* < 0.001, Figure 3J‐Q), showing a prolonged effect of SWS‐TMR when the cueing was halted. We further examined the changes of oscillatory events during the entire 4‐hour post‐learning NS, SWS or NREM (NS + SWS) in each group, and we only observed the significant increases of coupled spindles (*P* < 0.01) and coupled SOs (*P* < 0.001) in SWS‐TMR compared to the SWS substage in WN (Figure S5, Supporting Information), which further confirmed the prolonged effect of SWS‐TMR. Interestingly, the ratio of coupled spindle during total NREM sleep was positively correlated with the freezing response in the fear recall test in WN, SWS‐TMR, and NS‐TMR groups (*P* < 0.05, Figure S6, Supporting Information). Overall, our data indicates that TMR primarily modulates spindles by promoting the nesting of spindles within SOs in SWS‐TMR, which are key forces in driving memory reactivation and consolidation.^[^ ^46^, ^47^ ^]^ By analyzing sleep brain oscillations, we showed that the opposite effects of NS‐ and SWS‐TMR in fear memory consolidation were accompanied by the changes of spindle and SO‐spindle coupling.

### 2.4 Inhibition of Pyramidal Neurons in the Frontal Cortex Abolishes the Effects of TMR on Spindle Generation and SO‐Spindle Coupling

**Figure 4:**
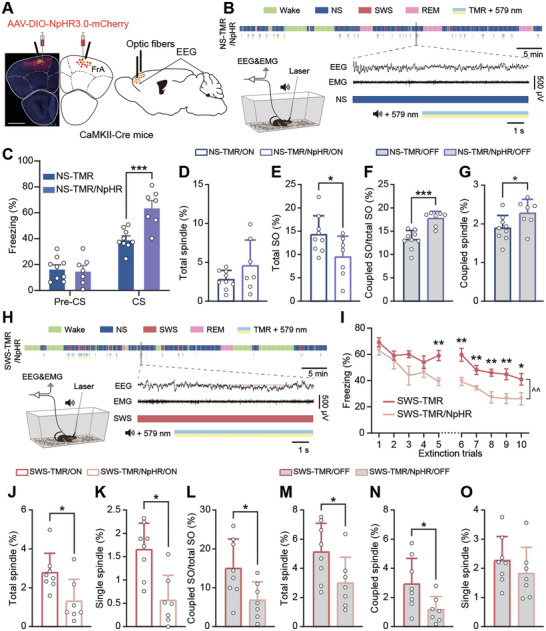
Optogenetic inhibition of the frontal cortex abolishes the effects of NS‐TMR and SWS‐TMR. A) Schematic diagrams and confocal image showing the locations of viral expression and optic fiber cannulas implantation in the FrA; scale bar, 1 mm. B) Example of sleep stages and CS tone presentations with optogenetic inhibition in NS‐TMR/NpHR. C) Freezing response in recall test during the pre‐CS and CS at Day 3. Data shown as mean ± SEM. NS‐TMR, *n* = 9, NS‐TMR/NpHR, *n* = 7. D–G): Quantification of time of oscillation subtypes in NS. Data shown as mean ± SD. NS‐TMR/ON, *n* = 9, NS‐TMR/NpHR/ON, *n* = 7, NS‐TMR/OFF, *n* = 9, NS‐TMR/NpHR/OFF, *n* = 7. H) Example of sleep stages and CS tone presentations with optogenetic inhibition in SWS‐TMR/NpHR. I) Freezing response in extinction trials during CS at Day 4 and 5. Data shown as mean ± SEM. SWS‐TMR, *n* = 9, SWS‐TMR/NpHR, *n* = 7. J–O): Quantification of time of oscillation subtypes in SWS. Data shown as mean ± SD. SWS‐TMR/ON, *n* = 8, SWS‐TMR/NpHR/ON, *n* = 7, SWS‐TMR/OFF, *n* = 8, SWS‐TMR/NpHR/OFF, *n* = 7. *P < 0.05, ***P < 0.001. For statistical analysis, data sets with normality and homogeneity of variance use two‐tailed unpaired *t*‐test; with normality and without homogeneity of variance use unpaired *t*‐test with Welch's correction; without normality use Mann Whitney test. For (I): ^^*P* < 0.01, two‐way ANOVA was used for comparison between SWS‐TMR and SWS‐TMR/NpHR groups over 10 trials.

During sleep, cerebral cortex plays a key role in orchestrating the information flow during memory consolidation, via neocortical slow oscillation, thalamocortical spindle, and hippocampal ripple.^[^ ^7^, ^48^ ^]^ However, it is unknown whether the frontal cortex plays a causal role in realizing the TMR effects and the associated neural oscillatory patterns. Therefore, we performed optogenetic inhibition of pyramidal neurons in the FrA by expressing Cre‐dependent halorhodopsin adenoassociated virus (AAV) in CaMKII‐Cre mice (**Figure** **4**A). Optogenetic inhibition was only administered simultaneously with the CS presentation in TMR (Figure 4B,H). Our data showed that the inhibition of the FrA during NS‐TMR (NS‐TMR/NpHR) abolished the memory impairment effect of NS‐TMR, such that mice showed higher freezing responses than the NS‐TMR group (*P* < 0.001, Figure 4B,C). Spindle generation was suppressed during the period that FrA was optogenetic inhibited with TMR cueing, which is similar to the effect of NS‐TMR without FrA inhibition (Figure 4D). However, NS‐TMR with FrA inhibition further significantly reduced the duration of total SOs and single SOs compared to NS‐TMR/ON (*P* < 0.05, Figure 4E; Figure S7A, Supporting Information), suggesting that the activity of pyramidal neurons in the FrA is important in SOs generation and SO‐spindle couplings in the NS stage. In contrast, upon cessation of NS‐TMR and optogenetic inhibition, we noted an increased proportion of coupled SOs (*P* < 0.001, Figure 4F) and duration of coupled spindles (*P* < 0.05, Figure 4G), without changes in the total spindles and SOs (Figure S7B, Supporting Information), indicating a higher propensity for spindles to occur during SOs. As a result, the ratio of coupled SOs over total SOs significantly increased while NS‐TMR and optogenetic inhibition halted (Figure 3F), and during the whole NS and NREM sleep (Figure S8, Supporting Information). Our data suggest that the homeostatic‐like rebound of SO‐spindle couplings observed during the OFF period of NS‐TMR with optogenetic inhibition could be related to the neutralization of fear memory impairment effects caused by NS‐TMR.

On the other hand, the inhibition of the FrA during SWS‐TMR (SWS‐TMR/NpHR) abolished the fear memory enhancement effect observed in fear extinction (Figure 4H,I, Figure S9, Supporting Information). When CS was played with the optogenetic inhibition of FrA during SWS, we observed significantly declined durations of total spindles and single spindles (*P* < 0.05, Figure 4J,K; Figure S10A, Supporting Information). As SWS‐TMR also showed prolonged effect for facilitating spindle generation during OFF period (Figure 3J‐Q), here we further found significantly lower levels of spindles (*P* < 0.05, Figure 4M,O; Figure S10B, Supporting Information), as well as SO‐spindle couplings (*P* < 0.05, Figure 3L,N) when SWS‐TMR and optogenetic inhibition halted, compared to the OFF period of SWS‐TMR. These findings suggest that the inhibition of pyramidal neurons in FrA during SWS suppresses the generation of spindles in a prolonged manner. Since the generation of spindles from the thalamus are believed to be triggered by cortical activities,^[^ ^48^, ^49^, ^50^ ^]^ our data suggest the involvement of the FrA in spindle generation, and the effects of TMR in memory consolidation.

### 2.5 Selective Reactivation of Fear Memory Associated Neurons in SWS‐TMR

**Figure 5:**
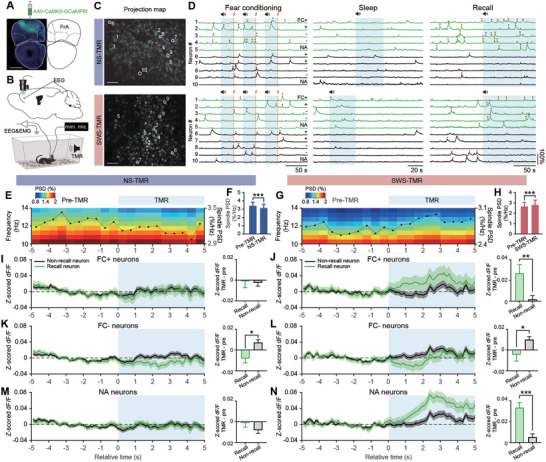
Calcium activities of sub‐populations of neurons induced by TMR. A) Schematic diagram and confocal image showing the location of viral expression; scale bar, 1 mm. B) Schematic diagram showing the locations of calcium imaging by miniature microscope and EEG electrode implantation. C) Representative calcium images; scale bar, 100 µm. D) Example calcium traces of neurons during fear conditioning, TMR in sleep, and recall test. Blue shadings denote CS tone presentation, red lines denote electric shock and red dots denote spiking burst events. Neurons are defined as FC positive (FC+), FC negative (FC‐), and not active (NA) depending on the neuronal spiking at different phases of fear conditioning; see methods. E–G): Power spectrogram of the 5 s before and after TMR (CS) onset. Dash lines with solid dots show the averaged spindle PSD for every 0.5‐second, scale on the right y‐axis. F,H): Quantification of the PSD in spindle band (10–14 Hz) during pre‐TMR (5 s) and TMR (first 5 s) periods. Data shown as mean ± SEM. *** P < 0.001, NS‐TMR trials = 235, mouse = 5, SWS‐TMR trials = 144, mouse = 4, mixed‐effects model. I–N): Traces show the z‐scored dF/F (minus mean dF/F of pre‐TMR) during the 5 s before and after TMR onset; data were shown as mean ± SEM. Bar charts show the quantification of changed dF/F between TMR (first 5 s) and pre‐TMR (5 s); data shown as mean ± SEM. *P < 0.05, **P < 0.01, *** *P* < 0.001. NS‐TMR: Mice = 5, TMR trials = 235, FC+_recall_ neurons = 151, FC+_non‐recall_ neurons = 512, FC‐_recall_ neurons = 305, FC‐_non‐recall_ neurons = 800, NA_recall_ neurons = 268, NA_non‐recall_ neurons = 608; SWS‐TMR: Mice = 4, TMR trials = 144, FC+_recall_ neurons = 137, FC+_non‐recall_ neurons = 566, FC‐_recall_ neurons = 274, FC‐_non‐recall_ neurons = 854, NA_recall_ neurons = 259, NA_non‐recall_ neurons = 670, mix‐effects model. The fixed effects and random effects for each model are listed in Table S1 (Supporting Information).

During sleep, reactivation of neuronal activities implicating in daytime learning during sleep was believed to support memory consolidation.^[^ ^51^, ^52^ ^]^ However, it is unknown if and how re‐presenting CS in TMR would modulate the reactivation of memory associated neurons, thereby facilitating or inhibiting memory consolidation. A recent study showed that neuronal responses in the L2/3 pyramidal neurons in the FrA integrate auditory cues and BLA inputs non‐linearly in a NMDAR‐dependent manner in fear learning.^[^ ^31^ ^]^ Here, we performed single cell‐resolution Ca^2+^ imaging on L2/3 pyramidal neurons using head‐mounted miniature microscope and EEG/EMG recording on freely behaving mice to examine the neuronal Ca^2+^ response during fear conditioning and fear recall in the FrA. (**Figure** **5**A,B). To trace the activity of individual neurons, GCaMP6f was expressed in neurons in the FrA by AAVs. Neurons were classified based on the present of spiking burst events (with at least 10 action potentials inferred from calcium data)^[^ ^53^, ^54^ ^]^ at different phase of FC: FC+ (with events at the late phase), FC‐ neurons (with events only at early phase) (see methods, Figure 5C,D). Neurons that did not fall into these two categories were characterized as “not active” (NA) neurons. Furthermore, based on their spiking burst events on Day 3 fear recall test, these neurons were further characterized as recall neurons (also known as neuron consolidation; green traces) and non‐recall neurons (black traces) (see methods; Figure 5D).

In human TMR studies, cue‐elicited spindle power was positively correlated with memory performance in spatial learning^[^ ^55^ ^]^ and vocabulary association learning tasks.^[^ ^15^, ^56^ ^]^ To examine the immediate effects of TMR (CS) on the EEG spindle PSD and the change of neuronal activity (z‐scored dF/F), we aligned all TMR trials within the 5‐second of pre‐TMR and the 5‐second after TMR onsets in a time‐locking manner (Figure 5E‐N). Regarding spindle activity, we found that spindle PSD was significantly reduced in NS‐TMR, but significantly increased in SWS‐TMR after the TMR onsets (*P* < 0.001, Figure 5F,H). When comparing the entire TMR/ON period and TMR/OFF period, NS‐TMR/ON spindle PSD was significantly lower than TMR/OFF period (*P* < 0.05, Figure S11A, Supporting Information); while there was no significant difference between the TMR/ON and TMR/OFF in SWS‐TMR (Figure S11B, Supporting Information). These results were consistent with the spindle oscillation data in Figure 3 (Figure 3H,P), in which the NS‐TMR exerted transient effect during TMR ON period and SWS‐TMR exerted prolonged effect on spindle generation. In addition, we found that the changes of spindle PSD toward TMR also positively correlated with freezing response in recall test at Day 3 (Figure S12, Supporting Information), suggesting that the modulation of spindle PSD during sleep directly impacts fear memory consolidation and animal freezing response.

Regarding the neuronal activity, we examined the immediate effects of TMR on calcium activity in different neuronal sub‐populations (FC+, FC‐, and NA) in respect to their reactivation during recall test (recall versus non‐recall neurons). Here we found that CS in NS‐TMR did not significantly affect neuronal activities in any neuronal sub‐populations comparing between Pre‐TMR and TMR period, but SWS‐TMR significantly induced higher dF/F in FC+_recall_, FC‐_non‐recall_ and NA_recall_ neurons (*P* < 0.01, Figure S13, Supporting Information). Furthermore, the enhancement of FC+ and NA neuron activities in SWS‐TMR was significantly more pronounced in recall neurons compared to non‐recall neurons (*P* < 0.01, Figure 5J,N). Since FC+ neurons intensively spiked during the last tone‐shock pairing of fear conditioning, indicating fear acquisition, we considered FC+ neurons as fear memory associated neurons. With trend analysis in each second, we further found that in SWS‐TMR, only FC+_recall_ neuronal activities were immediately increased (within 1 s, *P* < 0.05), whereas activities of other sub‐populations were sequentially increased in the latter time window of TMR (Figure S14, Supporting Information). These data suggest that SWS‐TMR triggers the reactivation of fear memory associated neurons immediately after the TMR onsets, followed by the increasing activities of other neuronal sub‐populations. On the contrary, in NS‐TMR, there was no significant increasing trend induced (within 1 s) by TMR in NS, nor any increasing trends during the following 5 seconds (Figure S14, Supporting Information). External CS presentation during sleep has been widely found to generate the cortical reactivation.^[^ ^57^ ^]^ Notably, our findings parallel with a recent human study examining TMR elicited scalp EEG: there were two temporally segregated neural representations following memory cues,^[^ ^58^ ^]^ which is similar to the two surges of neuronal activities in SWS‐TMR (Figure S14, Supporting Information). These data further demonstrate that SWS‐TMR immediately and selectively induces reactivation of fear memory associated neurons in the FrA.

Next, we applied the linear mixed‐effects model to analyze the correlation of dynamic changes between the spindle power and the neuronal activity of neuronal sub‐populations. Here we found that consolidated fear memory associated neurons (FC+_recall_ neurons) positively correlated with spindle PSD in SWS‐TMR (*P* < 0.01, *R* = 0.851, Figure S15B, Supporting Information), whereas the NS‐TMR group showed a negative correlation between spindle PSD and the FC+_non‐recall_ neurons (i.e., neurons that were not responsive during recall, *P* < 0.05, *R* = −0.698, Figure S15A, Supporting Information). Considering that thalamo‐cortical spindles contribute to memory consolidation,^[^ ^7^ ^]^ our data further suggest that only CS during SWS facilitates the cortical‐thalamic coordination via reactivating the consolidated fear memory associated neurons in the FrA.

### 2.6 Neuronal Response during TMR Predicts Consolidation of Memory Associated Neurons

**Figure 6:**
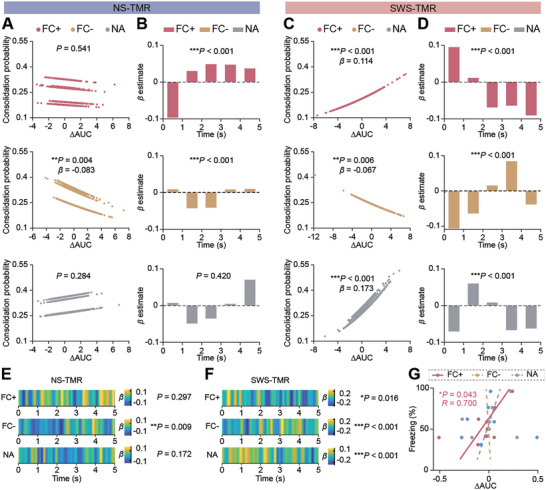
Neuronal response during TMR predicts consolidation of memory associated neurons. A,C): Generalized linear mixed‐effects models show the relationship between the change of area under curve of z‐scored dF/F (ΔAUC) during the first 5‐second of TMR presentation and consolidation probability (binomial, 1 for recall and 0 for non‐recall). Each dot represents the measurement of ΔAUC of one neuron in one TMR trial. *P*‐value for the fitted mixed‐effects model, *β* for the coefficient of fixed‐effect (ΔAUC during 5‐second TMR). NS‐TMR: FC+, measurements = 29905, neurons = 663, mice = 5; FC−, measurements = 50532, neurons = 1105, mice = 5; NA, measurements = 40076, neurons = 876, mice = 5. SWS‐TMR: FC+, measurements = 25424, neurons = 703, mice = 4; FC−, measurements = 40706, neurons = 1128, mice = 4; NA, measurements = 33550, neurons = 929, mice = 4. B,D): The coefficient *β* of ΔAUC during each second in generalized linear mixed‐effects models fitting the relationship between ΔAUC in each second and consolidation states. *P*‐value for the fitted mixed‐effects model. E,F): Heatmap showing the coefficient *β* of ΔAUC in each 0.1 second in generalized linear mixed‐effects models fitting the relationship between ΔAUC in each 0.1 s and consolidation states. *P*‐value for the fitted mixed‐effects model. The fixed effects and random effects for each model are listed in Table S1 (Supporting Information). G) Correlation between the mean ΔAUC during the first 5‐second of TMR and freezing rate in recall test at Day 3 (*n* = 9, including data from both NS‐TMR and SWS‐TMR). Linear regression was used to plot the line and two‐tailed Spearman correlation test was used for analysis. Only fitting model with *P* < 0.05 is showed in solid line, and the rests are showed in dashed lines.

As TMR differentially influenced FC+, FC‐, and NA neurons during NS and SWS, we next asked if the TMR‐induced neuronal activities would predict their consolidation fate (i.e., whether these neurons would be reactivated during Day 3 recall test or not). To address this question, we measured the change of dF/F (Δ area under curve, ΔAUC) during the 5‐second period following the onset of TMR in 5‐second (**Figure** **6**A,C), 1‐second (Figure 6B,D) and 0.1‐second (Figure 6E,F) resolution. By using generalized linear mixed‐effects models with ΔAUC in different neuronal sub‐populations, we generated regression models to describe the relationship between neuronal ΔAUC and the corresponding consolidation fate in each sub‐population (Figure 6A‐F). We first found that the overall ΔAUC (over 5 s) was positively correlated with consolidation probability in FC+ (*P* < 0.001, *β* = 0.11) and NA neurons (*P* < 0.001, *β* = 0.17) of SWS‐TMR (Figure 6C), indicating that in the SWS, FC+ and NA neurons with higher TMR‐induced neuronal activities are more likely to be reactivated during later memory retrieval. By contrast, TMR‐induced activity impeded the consolidation of FC‐ neurons in both NS‐TMR (*P* < 0.01, *β* = −0.08) and SWS‐TMR (*P* < 0.01, *β* = −0.07, Figure 6A,C). Furthermore, in the regression model with 1‐second resolution (Figure 6B,D), only FC+ neuron showed a positive coefficient (*β* estimate) in the first second after TMR onset of SWS‐TMR (Figure 6D), indicating the immediate TMR‐induced neuronal firing was instrumental for the consolidation fate of fear memory associated neurons. On the other hand, the initial increase of activity driven by TMR only positively but slightly impacted FC‐ neuron's consolidation fate in NS‐TMR (Figure 6B). Additionally, the 0.1‐second resolution models further verified that the activities immediately induced by SWS‐TMR facilitated the consolidation of FC+ neurons, while NA neurons were more likely to become consolidated neurons if their activities were induced during the 1–2 s time window post‐TMR onset (Figure 6F). Given that ΔAUC selectively influenced the consolidation fate of neuronal sub‐populations, we next ask whether ΔAUC also affects fear memory consolidation as shown in animal freezing response in the fear recall test. Interestingly, we found that only FC+ neurons in both SWS‐TMR and NS‐TMR groups showed a significant and positive correlation between the 5‐second ΔAUC and freezing response (*P* < 0.05, *R* = 0.70, Figure 6G), indicating that the higher increase of memory associated neurons activity induced by TMR corresponds to a more robust memory consolidation effect.

## 3 Discussion

Our findings provide novel and compelling evidence that NREM sleep constitutes a critical time window to selectively manipulate fear memory during sleep. This is the first report demonstrating that TMR during different substages of NREM sleep (NS similar to N1/N2; SWS, similar to N3 in human sleep) has opposite effects on fear memory consolidation, which are accompanied by changes in dendrite spine plasticity, spindle PSD, SO‐spindle coupling, and selective activation of fear memory associated neurons in the FrA. Optogenetic data further highlight the integral role of the FrA in the generation of spindles in both NS and SWS, which may facilitate cross‐regional information flow between cortical and subcortical brain regions for spindle generation and memory consolidation during NREM sleep.^[^ ^4^, ^8^ ^]^

For TMR experiment in rodents, it is crucial to consider the potential confounding effects of cue presentation during inappropriate sleep stages, as rodent sleep is more fragmented and shorter in duration in each sleep stage compared to human.^[^ ^4^ ^]^ Moreover, EMG activity during quiet wakefulness (QW) is similar to the EMGs in sleep stages, making it challenging to differentiate between these states in rodents.^[^ ^59^, ^60^ ^]^ Furthermore, unlike humans, rodents do not exhibit dominant alpha activity during QW, complicating the distinction between QW and sleep.^[^ ^60^ ^]^ To avoid delivering TMR cues during the wake‐to‐sleep transition, previous studies have opted to perform TMR only after a period of stable sleep.^[^ ^21^, ^22^ ^]^ In the current study, we similarly applied NS‐TMR after at least 20 s of NS stage identification (see methods). The power spectrum and Delta/Theta ratio analysis confirmed that NS‐TMR and SWS‐TMR were presented during NS and SWS, respectively, with the Delta/Theta ratio significantly different from active wakefulness (AW), QW, or REM sleep (Figure S16, Supporting Information).^[^ ^59^, ^61^, ^62^ ^]^

In the present study, our data showed that the time windows for fear memory editing in different substages of NREM sleep is critical: the SWS‐TMR can enhance fear memory within 24 h post‐learning sleep, while NS‐TMR fear attenuation effect is limited within the first few hours of post‐learning sleep. The fear memory‐facilitating effect of SWS‐TMR is consistent with prior findings,^[^ ^22^ ^]^ while the fear memory impairment of TMR after 24 h post‐fear learning has not been reported. Nonetheless, a recent study demonstrated that disruption of hippocampal structural long‐term potential within 4 h post‐learning sleep impaired contextual fear memory, but not in 4 h post‐learning wake period or 24 h post‐learning sleep period.^[^ ^63^ ^]^ Indeed, the effects of NS‐TMR within 4 h post‐learning sleep is different from a typical extinction in wakefulness. Previous studies showed that fear extinction in wakefulness immediately or within 30 min to 6 h after fear conditioning do not attenuate fear responses in later fear retrieval sessions.^[^ ^64^, ^65^, ^66^, ^67^ ^]^ These findings suggest that the fear attenuating effects of NS‐TMR were due to the modulation of memory consolidation during sleep within few hours after fear acquisition, instead of being a typical extinction in wakefulness. Together with our results, these findings reveal a limited time window for TMR‐induced fear memory impairment during sleep.

Memory reactivation during sleep requires the coordination of multiple brain regions, and spindle is believed to aid the thalamic‐cortical cross‐regional communication, among other regions.^[^ ^41^, ^49^, ^68^ ^]^ A previous study has demonstrated the important role of spindle to promote memory consolidation through triple phase‐locking of cortical, thalamic and hippocampal rhythms.^[^ ^7^ ^]^ Selective activation of memory associated neurons during SO‐spindle coupling phase has also been found to facilitate memory consolidation.^[^ ^45^ ^]^ Here, our data not only verify that SWS‐TMR promotes SO‐spindle couplings, but further demonstrate that the modulation of spindle is correlated with the TMR‐induced activity of memory associated neurons, which is also correlated with memory performance. Interestingly, we also found that the increase of neuronal activity induced by CS in FC‐_non‐recall_ neurons was significantly higher than in FC‐_recall_ neurons for both NS‐TMR and SWS‐TMR (Figure 5K,L). Since FC‐ neurons intensively spiked at the early phase but not the late phase of the fear conditioning, these neurons might not be responsible for integration and association of fear learning‐related information. Indeed, the higher activities of FC‐ neurons induced by TMR, the lower probability of the neurons to be consolidated (Figure 6A,C). These data raised an intriguing possibility: TMR during sleep not only promotes the reactivation of the fear memory associated neurons but might also actively eliminate the information with less‐importance.

Changes in the dendritic spine plasticity of L5 pyramidal neurons and the selective calcium responses of L2/3 pyramidal neurons in the FrA have been observed in both NS‐ and SWS‐TMR. Pyramidal neurons in L5 exhibit a unique dendritic architecture that features a prominent apical dendrite extending to the superficial layer, as well as a basal dendritic tree.^[^ ^69^, ^70^ ^]^ This structure enables L5 pyramidal neurons receive multiple inter‐cortical and cortico‐thalamic inputs at L1 and intra‐cortical inputs primarily from L2/3 pyramidal neurons.^[^ ^71^, ^72^, ^73^ ^]^ As the main output neurons of the cortical column, L5 pyramidal neurons project to a wide range of brain regions, such as thalamus.^[^ ^74^, ^75^ ^]^ Considering that the cortical inputs to TRN contribute to the generation of thalamic spindles,^[^ ^76^ ^]^ the direct projection of FrA to TRN^[^ ^37^ ^]^ (Figure S2, Supporting Information) further suggests that the generation of spindles in TRN could be induced by the activation of the FrA. A previous study showed that TRN neurons exhibit distinct activity patterns in slow wave and spindle.^[^ ^77^ ^]^ From our data, TMR during different NREM sleep substages show opposite influences in the generation of sleep spindles, suggesting that the TRN may have varying susceptibility to external stimuli in different sleep substages. Indeed, a recent study also proposed a model that convergent cortical slow wave downstates lead thalamic downstates, which triggers spindles that propagate back to the cortex.^[^ ^78^ ^]^ Interestingly, our data confirmed the correlation between the spindle power and consolidated memory associated neurons in L2/3 of the FrA (Figure S15B, Supporting Information), indicating the possibility that selective activation of memory associated neurons in the FrA caused by TMR during SWS could induce the spindle generation in the TRN. Consequently, this process might induce the synchronization of neuronal firings in selective circuits, resulting in formation and elimination of dendritic spines and synapses in a location‐specific manner in the L5 pyramidal neurons, facilitating memory consolidation.

A recent human TMR study observed that TMR induced two temporally segregated EEG activity: an immediate cue‐elicited EEG response and a subsequent, even stronger reactivation around 2‐second after CS.^[^ ^58^, ^79^ ^]^ The source analysis found that the second surge of EEG reactivation could be driven by the frontal cortex and hippocampal areas,^[^ ^58^ ^]^ and was associated with subseuqnet memory.^[^ ^79^ ^]^ Similarly, we also detected two surges of neuronal activities induced by SWS‐TMR: the first surge of FC+ neurons reactivation and a second surge of neuronal reactivation from both NA neurons and FC+ neurons in a delayed manner. Given that thalamic spindles spread to both cortical and hippocampal areas,^[^ ^49^, ^80^ ^]^ it is possible that the delayed neuronal activity surges in SWS‐TMR are induced by the coordination and feedbacks among thalamus, hippocampus, and the frontal cortex.

In contrast, the presentations of CS during NS (NS‐TMR) impaired fear memory. This raised the question of whether NS‐TMR functions through mechanisms similar to those involved in extinction during wakefulness. Fear extinction during wakefulness can be attributed to both “unlearning” and “new learning” mechanisms.^[^ ^81^, ^82^, ^83^ ^]^ The unlearning mechanism posits that extinction weakens the original fear memory trace, supported by evidence such as the depotentiation of synaptic inputs to the lateral amygdala during extinction^[^ ^84^, ^85^ ^]^ and the elimination of fear learning‐related or engram synapses post‐extinction.^[^ ^86^, ^87^ ^]^ Conversely, the new learning mechanism suggests that fear extinction represents the formation of a new inhibitory memory that suppresses the original fear memory, supported by the phenomenon of fear relapse^[^ ^88^ ^]^ and the existence of extinction‐specific engrams.^[^ ^89^, ^90^ ^]^ Indeed, the observed increase of dendritic spine formation in the FrA following NS‐TMR (Figure 1F) suggests the possibility of a “new learning” mechanism, similar to wakeful fear extinction. On the other hand, NS‐TMR might directly inhibit the normal memory consolidation process instead of acting through fear extinction mechanisms: unlike SWS‐TMR, we found that NS‐TMR inhibits SO‐spindle couplings and fails to trigger the reactivation of fear memory‐related neurons in the FrA. Notably, NS‐TMR has a limited working time window, i.e., within 4 h after fear conditioning. These findings suggest that NS‐TMR may interfere with the normal process of memory consolidation, as evidenced by its inhibition of consolidation‐related events (such as SO‐spindle couplings) and its loss of effectiveness once memory has been consolidated (24 h post‐conditioning). Given the fluctuations in EEG power spectrum and neuronal states during NREM sleep, it remains challenging to attribute the effects of NS‐TMR to a single mechanism and NS‐TMR might exert its effects on different brain regions via different mechanisms at different time points. Nonetheless, future study is needed to further examine the effects of TMR in other subcortical brain regions, such as thalamus, hippocampus, amygdala; as well as to examine the long‐term effects of NS‐TMR in fear reinstatement, renewal, and spontaneous recovery. In addition, it is important to test the potential use of TMR for fear memory editing during memory reconsolidation process in view of the limited time window of TMR‐induced fear impairment effect in the initial memory consolidation process.^[^ ^12^ ^]^

Taken together, our findings demonstrate the possibility of bidirectional manipulation of specific fear memory during different substages of NREM sleep. These findings not only provide new insights into the emotional memory consolidation and its accompanying neural mechanisms, but they also pave the ground for the future translation research in modifying pathological memory in psychiatric disorders, such as posttraumatic stress disorder.

## 4 Experimental Section

### Apparatus

Mice were trained and tested using the FreezeFrame system (Coulbourn Instruments). For fear conditioning, mouse test cages equipped with stainless‐steel shocking grids were connected to a precision feedback current‐regulated shocker (Coulbourn Instruments). For recall test and extinction, the shocking grids were replaced with non‐shocking test grids that differed in texture from the shocking grids used during conditioning. Each test cage was contained in a sound‐attenuating enclosure (Coulbourn Instruments). Behaviors were recorded using low‐light video camera (recording framerate: 5 Hz). Stimulus presentation was automated using Actimetrics FreezeFrame software (version 4.01; Coulbourn Instruments). All equipment was thoroughly cleaned with detergent followed by water between sessions.

### Auditory‐Cued Fear Conditioning

All fear conditioning experiments started at zeitgeber time 3±1. Mice were habituated in the chamber for 1 min (cage setup: shocking floor grids, ethanol scent). The fear conditioning (paired) was conducted with three pairings of a 30‐second, 12 kHz, 80 dB auditory cue (conditioned stimulus, CS) and a 2‐second 0.5 mA electric scrambled foot shock (unconditioned stimulus, US), which was administered in the last 2 s of the CS. There was a 15‐second intertrial interval. For the unpaired control group, mice received tones and shocks in an unpaired manner (tones and shocks were separated by random intervals of 5–15 s).

### Fear Recall Test and Fear Extinction

For the recall test, mice were placed in a different context (cage set‐up B: test floor grids, 1% Pinesol) for an initial 2‐min (pre‐CS) period and this was followed by tone presentation for 2 mins (CS). For extinction training, mice were subjected to five CS presentations (each lasting 2 mins with an intertrial interval of 2 mins) per day for two consecutive days.

### Animals

C57BL/6J, *CaMKIIα*‐Cre, and *Thy1*‐YFP (H line) mice were purchased from the Jackson Laboratory. Four‐ to six‐week‐old male *Thy1*‐YFP (H line) mice expressing YFP in pyramidal neurons were used in the dendritic spine imaging experiments. C57BL/6J mice were used for the in vivo Ca^2+^ imaging experiments. *CaMKIIα*‐Cre mice were used for the optogenetics experiments. Mice were bred and housed under an AAALAC International accredited program at the Centre for Comparative Medicine Research, HKU under Specific Pathogen Free conditions. Research only proceeded following review and approval from the HKU Committee on the Use of Live Animals in Teaching and Research (CULATR 5572‐20, 24‐181) and under license from the Hong Kong SAR Government's Department of Health. Mice were group‐housed in individually ventilated cages under a 12:12 dark light cycle within environmentally controlled rooms and were fed ad libitum with laboratory diet manufactured by LabDiet, USA.

### EEG/EMG Electrode Implantation

EEG/EMG electrodes were implanted as described previously.^[^ ^33^ ^]^ Briefly, mice were anaethetized with the isoflurane (3‐4% induction; 1.5‐2.5% maintenance at 0.5 L min^−1^ controlled by an animal anesthesia vaporizer RWD Life Science). Four electrodes were implanted to allow EEG/EMG recoding. Two electrodes were prepared for epidural EEG recording and another two electrodes were prepared for EMG recording. The electrode was made by the miniature pin connector (Miniature Pin Connector, 0.212 inch length, 0.032 inch width, A‐M Systems) soldering to the PFA‐coated silver wire (0.005 inch bare diameter, 0.007 inch coated diameter, A‐M Systems). Before the electrode implantation, a small area of the skull was thinned with a high‐speed drill and a small area of skull (≈0.2 mm in diameter) was removed by forcepts. The electrodes were bent at 1 mm from the tip of the silver wire and carefully inserted under the skull above the dura matter. One EEG electrode was placed over the left frontal cortex (2 mm lateral to midline, 2 mm anterior to bregma) and another on the cerebellum (at midline, 1 mm posterior of lambdoid suture), as depicted in Figure 1A. Two electrodes for EMG recording were placed on the nuchal muscle. The electrodes were fixed by cyanoacrylate‐based glue and further stabilized by dental cement. For dendritic spine imaging experiment, EEG/EMG surgeries were performed after cranial window implantation.

### EEG/EMG Recording and Sleep Staging

During sleep recording, EEG/EMG signals were recorded with a bandpass filter of 0.1‐100 Hz and the sampling rate was 10 kHz using BL‐420F data acquisition and analysis system (Chengdu Techman Co., Ltd) with the present of white noise generated by White Noise Machine (DOHM DS, USA) throughout the 4‐hour recording period. Online sleep staging were visually scored for the classification of sleep stages and wake status (Figure 1B,I). Briefly, the sleep was scored in every 5‐second epoch. Wake state was identified by lower amplitude and higher frequency of EEG activity, and medium to high muscle activity. REM sleep was identified by lower amplitude and higher frequency of EEG activity, and low muscle activity. In NREM epochs, an epoch would be further identified as SWS if there were two continuous synchronized, high‐amplitude slow wave appeared during the epoch. The rest of the NREM epochs were scored as non‐SWS (NS). For offine sleep analysis, the EEG/EMG signals were futher reviewed and the sleep stages were scored again. The accuracy of online sleep stage was further anlyszed offline (Figure S1C, Supporting Information).

### Quiet Wakefulness (QW) and Active Wakefulness (AW) Identification

To compare the difference between sleep stages and QW, ≈20 5‐second epoches of QW and AW respectively from each mouse were selected. Briefly, AW was identified by obvious high EMG activity in the 5 s. For QW, epochs were only selected that were flanked by the periods of active waking and with low EMG signal that is similar to the EMG in sleep stages, to avoid the periods of early transitional sleep identified as QW, as suggested by the previous study.^[^ ^59^ ^]^

### Delta/Theta Ratio Calculation

To compare the Delta/Theta ratio of different vigilance states,^[^ ^91^, ^92^ ^]^ 5‐second AW and QW epochs were selected as described above. REM sleep epochs with partial other stage were excluded. TMR epochs were the total period of each TMR administration, Pre‐TMR epochs were the 5‐second period before TMR onsets. The EEG of the epochs were first pre‐processed by a band‐pass filter (0.8 – 45 Hz) to remove potential noises, and then were used to perform power specturm analysis. Delta/Theta ratio = mean PSD in delta band / mean PSD in theta band.

### Targeted Memory Reactivation (TMR) Paradigm

Mice were randomly assigned to different groups. TMR was applied either immediately or 24 h after fear conditioning for 4 h during sleep in different experimental groups (Figure 2A). The conditioned stimulus (CS / Tone A, 12 kHz) or Tone B (4 kHz) was generated by a custom‐made Matlab (Mathworks) and Arduino system with a speaker on the top of cage. The tone playing time stamps were automatically recorded by Matlab. The tone was presented during sleep according to the previous study.^[^ ^21^ ^]^ Briefly, the presentation of tone (≈55 dB) lasted for at least 1 epoch (5 s) or for at most 6 epoch (30 s) each time and separated by at least 30 s. Presentation of tone was paused if the mouse awaken or the total of 5 min tone was given within 1 h. For NS‐TMR, tone was administered when at least 4 epochs of NS (20 s) were continuously identified. For SWS‐TMR, tone was administered once a synchronized, high‐amplitude slow wave sleep was identified.

### Cranial Window Implantation

Cranial window implantation was performed on *Thy1*‐YFP (H line) mice as described previously.^[^ ^34^ ^]^ Male mice (postnatal day 25 ± 1) were first anaesthetized with ketamine/xylazine (100 mg kg^−1^, 10 mg kg^−1^ respectively in PBS, i.p.). Before the surgery, mice were injected with carprofen (5 mg kg^−1^, s.c.) and dexamthasone (2 mg kg^−1^, i.m.). Scalp was removed and the tissues on the skull surface were carefully cleaned. A metallic head‐mount was fixed on the skull with glue and dental cement. A square craniotomy (2 mm × 2 mm) was created on the right frontal association cortex (FrA: +2.8 mm bregma, +1.0 mm midline) with high‐speed drill and forceps. Dura mater was removed under artificial cerebrospinal fluid (ACSF: 119 mM NaCl, 2.5 mM CaCl_2_, 1 mM NaH_2_PO_4_, 2.5 mM KCl, 1.3 mM MgSO_4_, 26.2 mM NaHCO_3_, and 22 mM glucose). Coverglass (#1 thickness, Electron Microscopy Sciences) was placed on the craniotomy window and carefully fixed by sealing with a layer of tissue adhesive (Vetbond, 3 M Company), and later with another layer of cyanoacrylate gel and dental cement. Finally, the EEG/EMG electrodes were implanted as described above. Carprofen (intraperitoneal injection, 5 mg kg^−1^) and buprenorphine (subcutaneous injection, 0.1 mg kg^−1^) were administered to reduce the inflammation and alleviate the pain from the surgery twice daily for five days.

### In Vivo Two‐Photon Imaging

Mice were subjected to at least 10 days of recovery after cranial window implantation before imaging experiment. During the imaging, mice were anaesthetized by ketamine/xylazine and head‐fixed by the head‐mount and a customized platform to minimize the motion during imaging. A two‐photon microscope (Olympus FVMPE‐RS) equipped with Coherent Chameleon Vision II lasers tunable from 680 to 1080 nm was used for imaging. Laser was set to 920 nm for imaging the YFP signal and a water immersion objective lens (25 ×, N.A. = 1.05, Olympus) was used. The z‐stack images of dendritic spines were then acquired in high resolution (0.14 µm per pixel XY resolution, 0.75 µm step size) with Galvo scanners.

### Virus Injection for Optogenetics

Mice (*CaMKIIα*‐Cre, P25 ± 1) were first anaethetized with the isoflurane (3‐4% induction; 1.5‐2.5% maintenance) at 0.5 L min^−1^ controlled by an animal anesthesia vaporizer and supplied to mice on the stereotaxic apparatus. AAV2/8‐EF1a‐DIO‐NpHR3.0‐mCherry‐WPRE (for halorhodopsin expression, titer 2.00 × 10^12^ genome copy (GC)/ml, BrainVTA) or AAV2/8‐EF1a‐DIO‐mCherry‐WPRE (for vector control, titer 2.00 × 10^12^ GC mL^−1^, BrainVTA) was diluted in ACSF (1:0.5, 400 nl) and bilaterally injected into the FrA (+2.8 mm bregma, +1.0 mm midline, +0.4 mm from surface of brain) at 100 nL min^−1^ through Neuros Syringes (Hamilton; 33G, blunt end) driven by KDS Legato 130 syringe pump (KD Scientific).

### Fiber Implantation

Dual fiber‐optic cannulas of 200 µm thickness and 0.39 NA spaced 1.25 mm apart (RWD Life Science) were implanted into the AAV injection sites with the stereotaxic instrument and holder right after the virus injection on each site. The optic fibers were fixed by tissue adhesive and dental centment. EEG/EMG electrodes were next implanted as described above. Carprofen (5 mg kg^−1^, i.p.) and buprenorphine (0.1 mg kg^−1^, s.c.) were administered to reduce the inflammation and alleviate the pain from the surgery twice daily for five days.

### Optogenetic Inhibition and TMR Experiment

Three weeks after the AAV injection and optic fibers implantation, mice were habituated with the attachment of optic fiber cables and EEG/EMG cables in clean cage for 1 hour daily, two continuous days before experiment. During the sleep recording, EEG/EMG was recored as described above. CS and 579 nm laser (20 mW on system, ≈4 mW measured from the optic fiber tip, 20 Hz, 50 ms pulse width, Aurora‐400, Newdoon) were administered simultaneously via a custom‐written Matlab and Arduino system.

### Virus Injection for Ca^2+^ Imaging

Male C57BL/6J mice were first anaethetized with the isoflurane (3‐4% induction; 1.5‐2.5% maintenance at 0.5 L min^−1^ controlled by an animal anesthesia vaporizer). AAV9.CaMKII.GCaMP6f.WPRE (titer 5.73 × 10^12^ GC/ml, BrainVTA) was diluted in ACSF (1:0.5, 400 nl) and injected into the right FrA (+2.8 mm bregma, +1.0 mm midline, +0.4 mm from surface of brain) at 100 nL min^−1^ through Neuros Syringes (Hamilton; 33G, blunt end) driven by KDS Legato 130 syringe pump (KD Scientific).

### Virus Injection for Projection Tracing

C57BL/6J mice were first anaethetized with the isoflurane (3‐4% induction; 1.5‐2.5% maintenance at 0.5 L min^−1^ controlled by an animal anesthesia vaporizer). AAV9.hSyn.TurboRFP.WPRE.bGH (titer 1.09 × 10^14^ GC mL^−1^, the University of Pennsylvania Vector Core) was diluted in ACSF (1:4, 100 nL) and injected into the right FrA (+2.8 mm bregma, +1.0 mm midline, +0.4 mm from surface of brain) at 100 nL min^−1^ through Neuros Syringes (Hamilton; 33G, blunt end) driven by KDS Legato 130 syringe pump (KD Scientific).

### Cranial Window and Baseplate Implantation for Miniature Microscope

Three weeks after virus injection, mice were subjected to cranial window, baseplate, and EEG/EMG implantation. Mice were anaesthetized with ketamine/xylazine (100 mg kg^−1^, 10 mg kg^−1^ respectively in PBS, i.p.). Before the surgery, mice were injected with carprofen (5 mg kg^−1^, s.c.) and dexamthasone (2 mg kg^−1^, i.m.). Scalp was removed and the tissues on the skull surface were carefully cleaned. A metallic head‐mount was fixed on the skull with glue and dental cement. A round craniotomy (4 mm × 4 mm) was created on the right frontal association cortex (FrA: +2.8 mm bregma, +1.0 mm midline) with high‐speed drill and forceps. Dura mater was removed under artificial cerebrospinal fluid (ACSF: 119 mM NaCl, 2.5 mM CaCl_2_, 1 mM NaH_2_PO_4_, 2.5 mM KCl, 1.3 mM MgSO_4_, 26.2 mM NaHCO_3_, and 22 mM glucose). Coverglass (# 1 thickness, Warner Instruments) was placed on the craniotomy window and carefully fixed by sealing with a layer of tissue adhesive (Vetbond, 3 M Company), and a layer of cyanoacrylate gel and Metabond. Next, the baseplate of the miniature microscope (nVoke 1.0, Inscopix, Palo Alto, CA) was placed above the glass window by aligning the best imaging focus of the neuronal calcium signals. The dental cement was added to secure the position. EEG/EMG electrodes were next implanted as described above. Carprofen (5 mg kg^−1^, i.p.) and buprenorphine (0.1 mg kg^−1^, s.c.) were administered to reduce the inflammation and alleviate the pain from the surgery twice daily for five days.

### In Vivo Ca^2+^ Imaging

Mice were subjected to at least 10 days of recovery after cranial window implantation. Mice were habituated with the attachment of miniature microscope and EEG/EMG cables for at least 1 h daily, two consecutive days before baseline recording. The Ca^2+^ imaging recording was performed during fear conditioning and recall test. Before each imaging session, the miniature microscope was fixed on the baseplate by tightening the screw. Images were acquired frame rate 20 Hz with 50 ms exposure time. The gain and power of LED were adjusted according to the qualtiy of calcium signal of each mouse. To alleviate the impact of the heat by illumination, the maximum time of each Ca^2+^ imaging recording was 20 min and all TMR events were recorded during sleep.

### Analysis of EEG/EMG Data

The offline analysis of EEG/EMG data and oscillatory events identification were performed by the custom‐written Matlab programs based on previous studies.^[^ ^7^, ^45^ ^]^

### Power Spectrum Density Analysis

Data was divided by scored sleep stages. Each part of EEG data was down sampled to 1000 Hz and bandpass filtered at 0.3‐45 Hz. The spectrum power density (PSD) was calculated by Welch's power spectrum density estimate with Hanning window tapering. The individual frequency band was defined as the delta band (0.8‐4 Hz), theta band (4‐8 Hz), alpha band (8‐14 Hz) and beta band (14‐30 Hz). The spindle band was defined as 10–14 Hz. The PSD of each band was calculated as the accumulated power in each frequency band (normalized by the total power) over the width of the band.

### Identification of Slow Oscillation (SO)

The EEG data was bandpass filtered at 0.3‐4.5 Hz. All the interval times between two successive positive to negative zero‐crossings were marked as oscillation events and the local amplitude between the peak and trough in each oscillation event was calculated. The threshold for the time interval between two continuous zero‐crossings was set as 0.4 to 2 s (corresponding to 0.5‐2.5 Hz). The candidate oscillation events were identified as the SO if the absolute amplitude of trough and the maximum to minimum amplitude of the candidate event were greater than 66.67% (top one‐third) respective amplitude values of all oscillation events according to the previous studies.^[^ ^7^ ^]^

### Identification of Spindle

The EEG data was bandpass filtered at 10–14 Hz. Hilbert transform was performed to create the envelope of the EEG data. The standard deviation (SD) of the envelope was calculated, and candidate spindle event would be marked if the envelope was greater than the 3.5 times of the SD. The onset and the end timepoints of each candidate spindle event were defined as the timepoints crossing the 2 times of the SD of the envelope. The thresholding duration for the individual spindle was set between 0.4 and 3 s.

### Identification of SO‐Spindle Coupling

After the identification of SO and spindle, the SO‐spindle coupling was determined when the time difference between the peak of spindle and the peak of SO was between −0.5 s and 1 s (meaning that spindle's peak precedes SO's peak within 0.5 s, or spindle's peak succeeds SO's peak within 1 s). In this case, the SO and the spindle were named coupled SO and coupled spindle. Accordingly, the rest SO and spindle were named single SO and single spindle in this study.^[^ ^45^ ^]^

### Quantification of Oscillation Events

The percentage of time of each identified oscillation event in a targeted sleep stage was calculated as the time of the oscillation event in the sleep stage divided over the total time of the sleep stage. The ratio of coupled SO / total SO was calculated as the time of the coupled SO divided over the sum of the time of coupled SO and single SO. Similarly, the ratio of coupled spindle / total spindle was calculated as the time of the coupled spindle divided over the sum of the time coupled spindle and single spindle.

### Clustering Analysis with Simulation

To verify the clustering feature of SWS epochs, a custom‐written Matlab program was used (Figure 1C). The simulation was performed based on the number of SWS epochs and the real timestamps of NS epochs, REM epochs and Wake epochs. The cumulative distribution function analysis of the closest intervals between each two SWS epochs was subsequently performed on the simulated data (1000 times of repeats) and real data. Two‐sample Kolmogorov‐Smirnov test was made between simulated data and real data on Matlab.

### Deep Learning Model for Classification of Sleep Stages

The deep learning model employed in this study was built upon the architecture from prior research.^[^ ^30^ ^]^ To prepare the inputs, electroencephalogram (EEG) data from each mouse was initially rescaled by computing the mean of the absolute values across the entire time duration. Each 5‐second epoch was then plotted and stored as graphical inputs, accompanied by their manually labeled stages. Subsequently, 20% of the data was randomly set aside as testing data, while the remaining data was utilized for model training. The model's accuracy was assessed by evaluating its performance on the held‐out testing data. In the confusion matrix (Figure 1K), the term “Predictions” refers to the sleep stages of the EEG epochs as determined by the deep learning‐trained model, while “True labels” represent the sleep stages as scored manually. A custom‐written Matlab program was used for the development and implementation of this model.

### Analysis of Dendritic Spine Imaging Data

All data analysis was performed blind to treatment conditions. All dendritic spine data were analyzed using FIJI software (ImageJ, National Health Institute), and the representative dendritic spine images were processed with Adobe Photoshop. The criteria to identify dendritic spines were according to the previous publication.^[^ ^34^ ^]^ Briefly, the dendritic segments with high image quality (ratio of signal to background noise >4:1) were selected. The number and location of the dendritic spines on each selected dendritic branch were manually quantified in the 3D image stack. The plasticity of dendritic spine (elimination and formation) was tracked across different time points on the same location of dendritic branch. The elimination or formation rate was calculated as the number of eliminated or formatted spines on the target time point divided by the number of spines on the previous time point. For Figure 2I, the relative distance between the location of eliminated spine identified at Day 3 and the location of newly‐formed spine identified at Day 5 were measured manually using FIJI. The maximum volume of the dendritic segments analyzed was 216 µm × 216 µm × 170 µm below the pial surface. The analysis of dendritic spine data was done blind to the experimental group of animals.

### Analysis of Ca^2+^ Imaging Data

All data analysis was performed blind to treatment conditions. All calcium imaging movies were processed using the Inscopix Data Processing Software (v1.6.0). The recording video files for one mouse were concatenated and processed as one single video (20 Hz, frame size 1440 × 1080 pixels equivalent to 900 × 650 µm). The pre‐procession was employed both spatial and temporal (4 times) and temporal (2 times) down‐sampling to balance the file size and accuracy. The pre‐processed videos were spatially bandpass filtered, with the adjusted parameters of the low cut‐off and the high cut‐off according to the video quality, to remove the low spatial frequency noise and smoothen the high spatial frequency components from the movies. Subsequent motion correction was performed to remove the frame‐to‐frame motion variations in the movies. Additionally, if the orientation of the recordings differed across experimental sessions, the videos were rotated by a specified angle to standardize the recording slope. To calculate the delta F/F (dF/F), an initial baseline frame (F0) was first computed by averaging the value of each pixel across the entire movie. Subsequently the delta F/F (dF/F) value of each frame of the movie was calculated as follows:

(1) $$M^{\prime }\left(x,y,t\right)=\frac{M(x,y,t)-F0(x,y)}{F0(x,y)}\forall \left(x,y,t\right)$$

where M(x,y,t) is the original value at pixel coordinate (x,y) of frame t of the movie, M’ is the output movie values, and F0(x,y) is the value of the calculated baseline frame at pixel coordinate (x,y). ∀(x,y,t) represents each pixel annotated through all corresponding units over the whole movie time. Due to the varying recording quality of the videos over days, the region of interest (ROI) of each neuron ensemble was identified by manual drawing contours on the processed movie according to the visual assessment.

The time‐stamped traces data of the identified neurons with dF/F was then generated for the calcium spiking burst event detection. The event detection algorithm was used in Inscopix Data Processing Software (v1.6.0) to automatically identify the neuronal events when bursts of cell activity occur. Based on the assumption that the calcium activity associated with an event should exhibit a monotonic rise, succeeded by an exponential decay, events were identified by a rapid increase in amplitude, followed by an extended decay returning to the baseline level. Specifically, the Median Absolute Deviation (MAD) was first calculated for the target cell trace.^[^ ^93^ ^]^ Subsequently, the trough values and peak values were identified corresponding to the transitions from positive to negative and negative to positive, respectively, within the cell trace. The current threshold value was computed as the preceding trough value plus the scaled MAD, which served as the thresholding criterion for event detection. When the current value of the cell trace exceeded the current threshold, we marked it as a potential event period and determined the time of peak during the potential event period. If the decay time from the peak to the end of the period exceeded the predefined decay time threshold, we considered it as a spiking burst event. Based on previous studies,^[^ ^53^, ^54^ ^]^ the decay time for a single action potential in GCaMP6f is around 150–200 ms, while the decay time for 10 action potential in GCaMP6f is approximately 400 ms. To better identify neurons exhibiting intense spiking events in each behavioral phase, the neurons were therefore selected with event decay times exceeding 400 ms for the detection of spiking burst events. In Figure 5D, the spiking burst events were identified during fear conditioning and fear recall, marked with red dots.

### Identification of Neuron Sub‐Populations

With the identification of spiking burst events during fear conditioning (FC) on Day 1, neurons were labeled as FC+ neurons, FC‐ neurons, and NA neurons according to spiking burst events occurred during early or late phases of FC: 1) Early phase: 1st tone, 1st shock with following 10 s, 2nd tone, 2nd shock with following 10 s; 2) Late phase: 3rd tone, 3rd shock with following 10 s. Neurons with spiking burst events only at early phase but not the late phase were marked as FC‐ neurons; whereas neurons with spiking burst events during the late phase were marked as FC+ neurons. Neurons without spiking burst events in these two periods were marked as “not applicable (NA)”. Neurons were further defined as recall and non‐recall neurons: 1) Recall neurons, neurons with spiking burst events during the 1^st^ minute of CS in the recall test; 2) Non‐recall neurons, neurons without spiking burst event during the 1^st^ minute of CS in the recall test. Therefore, FC+ and FC‐ neuron populations were further divided into FC+_recall_ neurons, FC+_non‐recall_ neurons, FC‐_recall_ neurons, and FC‐_non‐recall_ neurons, NA_recall_ neurons, NA_non‐recall_ neurons based on their spiking burst events during recall test.

To compare the neuronal traces data (dF/F), dF/F was further processed by z‐score normalization by subtracting the mean and dividing by the standard deviation of all neurons in the corresponding mouse. For the analysis of the pre‐ and the 5 seconds after the onsets of TMR, the neuronal trace of the change of dF/F (z‐score) was calculated as the trace data (z‐scored dF/F) minus the mean of the trace data during the pre‐TMR (5 s). The quantification of the change of dF/F was calculated as the mean of the trace data during the 5 s after the onsets of TMR minus the mean of the trace data during the 5 s pre‐TMR (Figure 5I‐N).

### Statistical Analysis

The GraphPad Prism software 9.0 (GraphPad Software) or Matlab with a significant level set at 0.05 was used to perform statistical analysis and create the statistical figures. Data are presented as the mean ± SD or mean ± SEM unless otherwise stated. The Shapiro–Wilk test was used to test the normality of all data sets. The *F*‐test was used to test for the homogeneity of variance of two groups while the Brown–Forsythe test was used to test for the homogeneity of variance of three or more groups. For comparison between two data sets that were measured from same corresponding individuals: paired *t*‐test was used when data sets are normal distribution; Wilcoxon matched‐pairs signed rank test was used when data sets are not normal distribution. For comparison between two data sets that were measured from two different groups: unpaired *t*‐test was used when data sets were normal distribution and with equal population variances; unpaired *t*‐test with Welch's correction was used when data sets were normal distribution and with unequal population variances; Mann Whitney test was used when data sets were not normal distribution. For comparison among all data sets from three or more groups, one‐way ANOVA followed by *post hoc* analysis using Tukey's multiple comparisons test was used when data sets were normal distribution and with equal population variances; Welch's ANOVA followed by *post hoc* analysis using Dunnett's T3 multiple comparisons test was used when data sets were normal distribution and with unequal population variances; Kruskal‐Wallis test followed by *post hoc* analysis using Dunn's multiple comparisons test was used when data sets were not normal distribution.

For the correlation analysis, Pearson's correlation test was used when data sets were normal distribution; Spearman correlation test was used when data sets were not normal distribution. For the trend test of neuronal activities, the Mann Kendall Trend test was used on the trace data during each 1‐second period to test if the trace is in an increase trend or decrease trend or not during each second. For the clustering analysis of SWS epochs, two‐sample Kolmogorov‐Smirnov test (using Matlab “kstest2”) was used between simulated data and real data. For the comparison of spindle PSD between Pre‐TMR and TMR and the analysis of the activities of neuronal subpopulations, the linear mixed‐effects model (using Matlab “fitlme”) was used. This model considers the data dependence for the repeated measurements from same animal or same trial when computing the statistical significance, and it is more stringent than simply computing it over all data.^[^ ^94^, ^95^ ^]^ Here, random intercepts were fitted for each comparison, and the random effects and reported *P* values for the model were listed (Table S1, Supporting Information).

For the regression modeling of changed neuronal activity (delta area under curve, ΔAUC) and consolidation states, the generalized linear mixed‐effects model (using Matlab “fitlme”) was used to estimate the consolidation probability given by its ΔAUC. The binomial distribution with logistic link function was used in the model. For Figure 6A,C, ΔAUC calculated as the difference between the entire 5‐second after TMR onset and the entire 5‐sec in pre‐TMR was used as the unique‐variable for fixed effect. For Figure 6B,D, ΔAUC calculated as the change during each 1‐ second period was used as the multiple‐variables for fixed effects of the model. For Figure 6E,F, ΔAUC calculated as the change during each 0.1‐ second period was used as the multiple‐variables for fixed effects of the model. The reported *P* value for each model and the coefficient for each variable of fixed‐effects were listed (Table S1, Supporting Information). Unless otherwise stated, statistical tests were two‐tailed, and *P* values less than 0.05 were considered as statistically significant.

## Conflict of Interest

The authors declare no conflict of interest.

## Author Contributions

Q.Z., X.H., and C.S.W.L. conceived and designed the research. Q.Z. and Y.H. conducted experiments. Q.Z. and C.M. contributed to the algorithm development for calcium imaging data. Q.Z. and C.S.W.L. analyzed data. C.S.W.L. and X.H. supervised the research. All contributed to the interpretation of the results. Q.Z. and C.S.W.L. wrote the original draft. Q.Z., Y.H., X.H., and C.S.W.L. reviewed and edited the manuscript.

## Supporting information

Supporting Information

Supplemental Table 1

## Data Availability

The data that support the findings of this study are available from the corresponding author upon reasonable request.
