# Supplementary material for: Selective Modulation of Fear Memory in Non‐Rapid Eye Movement Sleep
Source: Adv Sci (Weinh). 2024 Oct 9;11(44):2400662. doi: 10.1002/advs.202400662 (PMC11600212; doi:10.1002/advs.202400662)
Supplement: Supplementary file 1 — Supporting Information [file ADVS-11-2400662-s002.docx]

Supporting Information

Selective Modulation Of Fear Memory In Non-Rapid Eye Movement Sleep

Qiyu Zheng, Yuhua Huang, Changrui Mu, Xiaoqing Hu*, Cora Sau Wan Lai*


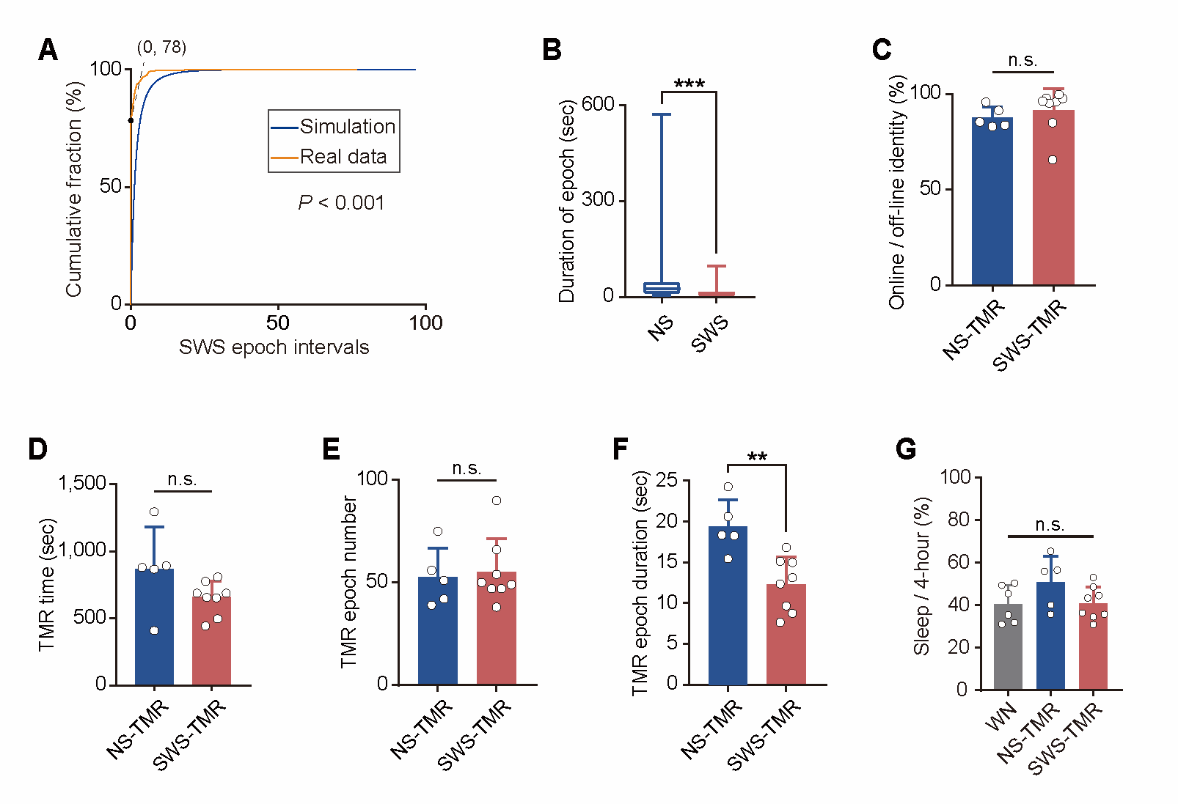


**Figure S1.**

(**A**) Clustering analysis of SWS epochs showing the intervals between the closest two epochs in real data and simulation data (shuffled 1000 times). Real data versus simulation, *P* < 0.001, two-sample Kolmogorov-Smirnov test. Each epoch lasts 5 seconds. Point (0, 78) shows that 78% of SWS epochs are preceding or following by another SWS epoch. (**B**) Quantifications of the duration in each continuous NS or SWS epoch. NS, *n* = 1557, SWS, *n* = 1308; data shown as box-whisker plots with box limits representing the ﬁrst and third quartile and whiskers indicating the data range. (**C**) to (**F**): Quantifications of the properties of NS-TMR and SWS-TMR. NS-TMR, *n* = 5, SWS-TMR, *n* = 8. Data shown as mean ± SD. ***P* < 0.01, ****P* < 0.001, n.s., not significant; data sets with normality and homogeneity of variance use two-tailed unpaired *t*-test; with normality and without homogeneity of variance use unpaired *t*-test with Welch's correction; without normality use Mann Whitney test. (**G**) Propotion of sleep time over the 4-hour recording period. WN, *n* = 6, NS-TMR, *n* = 5, SWS-TMR, *n* = 8. Data shown as mean ± SD. n.s., not significant. One -way ANOVA, Tukey's multiple comparisons *post hoc* test.


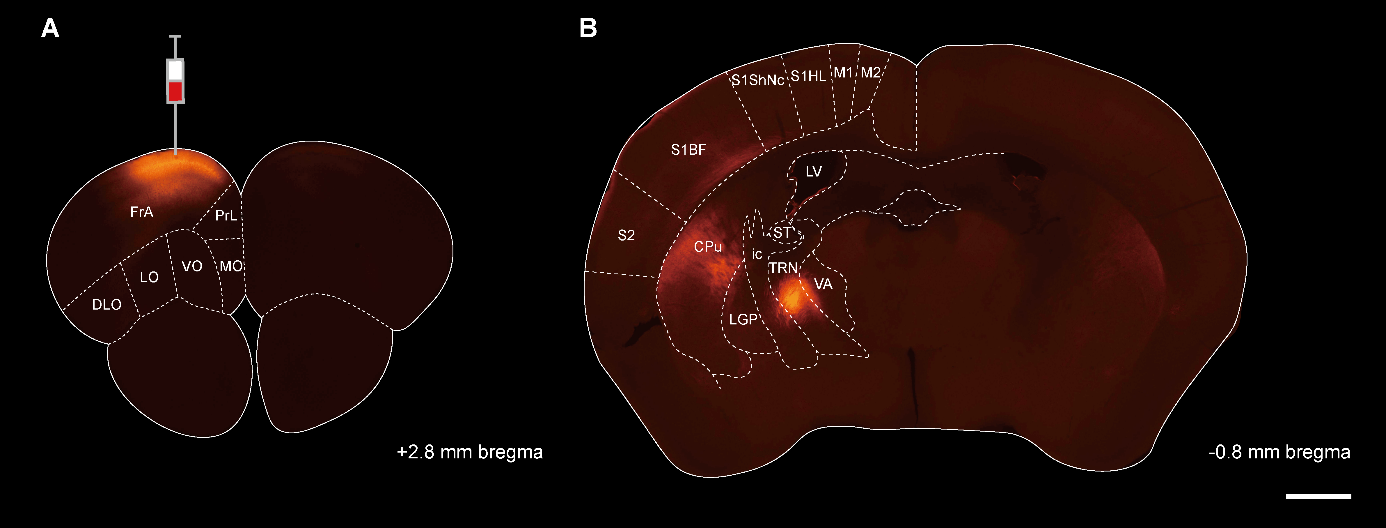


**Figure S2.**

(**A**): Injection site of adeno-associated virus (AAV-hSyn-TurboRFP) in the frontal association cortex (FrA) for anterograde projection labeling (at bregma +2.8 mm). (**B**) : An image of labeled axonal projections in the thalamic reticular nucleus (at bregma -0.8 mm). PrL: prelimbic cortex; MO: medial orbital cortex; VO: ventral orbital cortex; LO: lateral orbital cortex; DLO: dorsolateral orbital cortex; TRN: thalamic reticular nucleus; VA: ventral anterior thalamic nucleus; ST: stria terminalis; LV: lateral ventricle; ic: internal capsule; LGP: lateral globus pallidus; CPu: caudate putamen (striatum); S2: secondary somatosensory cortex; S1BF: primary somatosensory, barrel; S1ShNc: primary somatosensory, shoulder and neck; S1HL: primary somatosensory, hindlimb; M1: primary motor cortex; M2: secondary motor cortex.


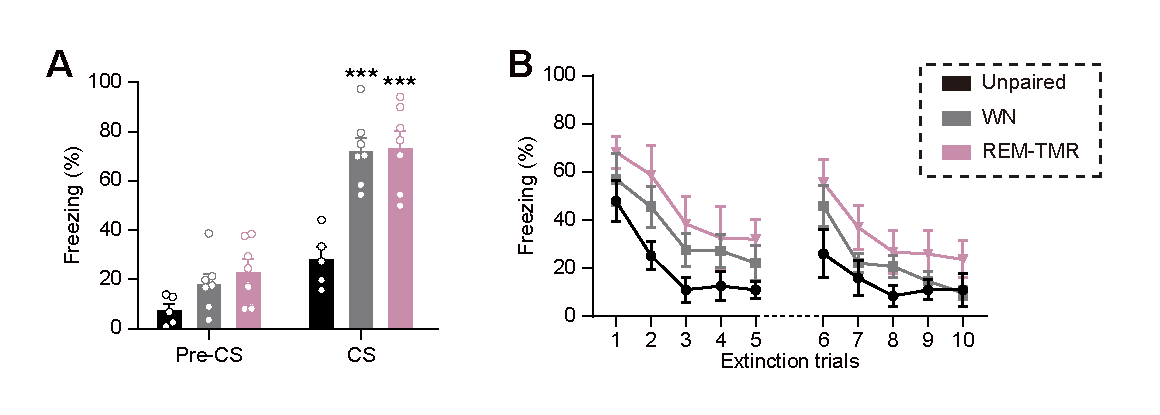


**Figure S3.**

(**A**) Freezing response in recall test during the pre-CS and CS at Day 3. (**B**) Freezing response in extinction trials during CS at Day 4 and 5. Data shown as mean ± SEM. Unpaired, *n* = 5, WN, *n* = 7, REM-TMR, *n* = 7; ****P* < 0.001; marked group compared to Unpaired; for (A), one-way ANOVA, Tukey's multiple comparisons *post hoc* test; for (B), two-way ANOVA was used for comparison over 10 trials.


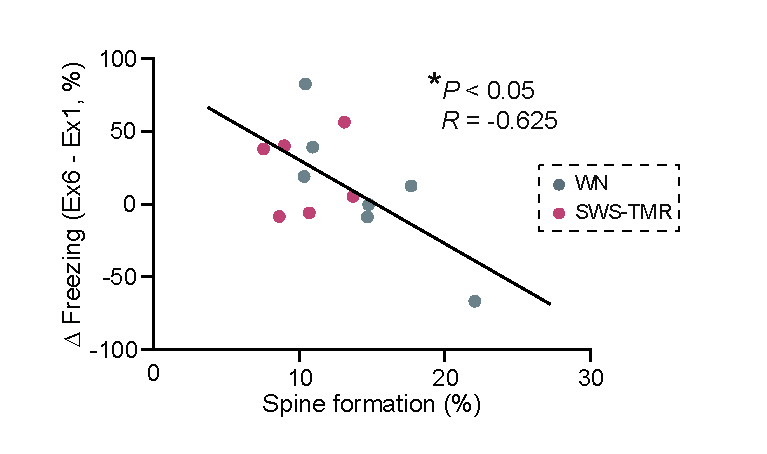


**Figure S4.**

Correlation between spine formation induced by fear extinction at Day 5 and the changed of freezing rate between the first extinction trial at Day 5 (Ex6) and the first extinction trial at Day 4 (Ex1); linear regression was used to plot the line, and two-tailed Pearson’s correlation test was used for analysis.


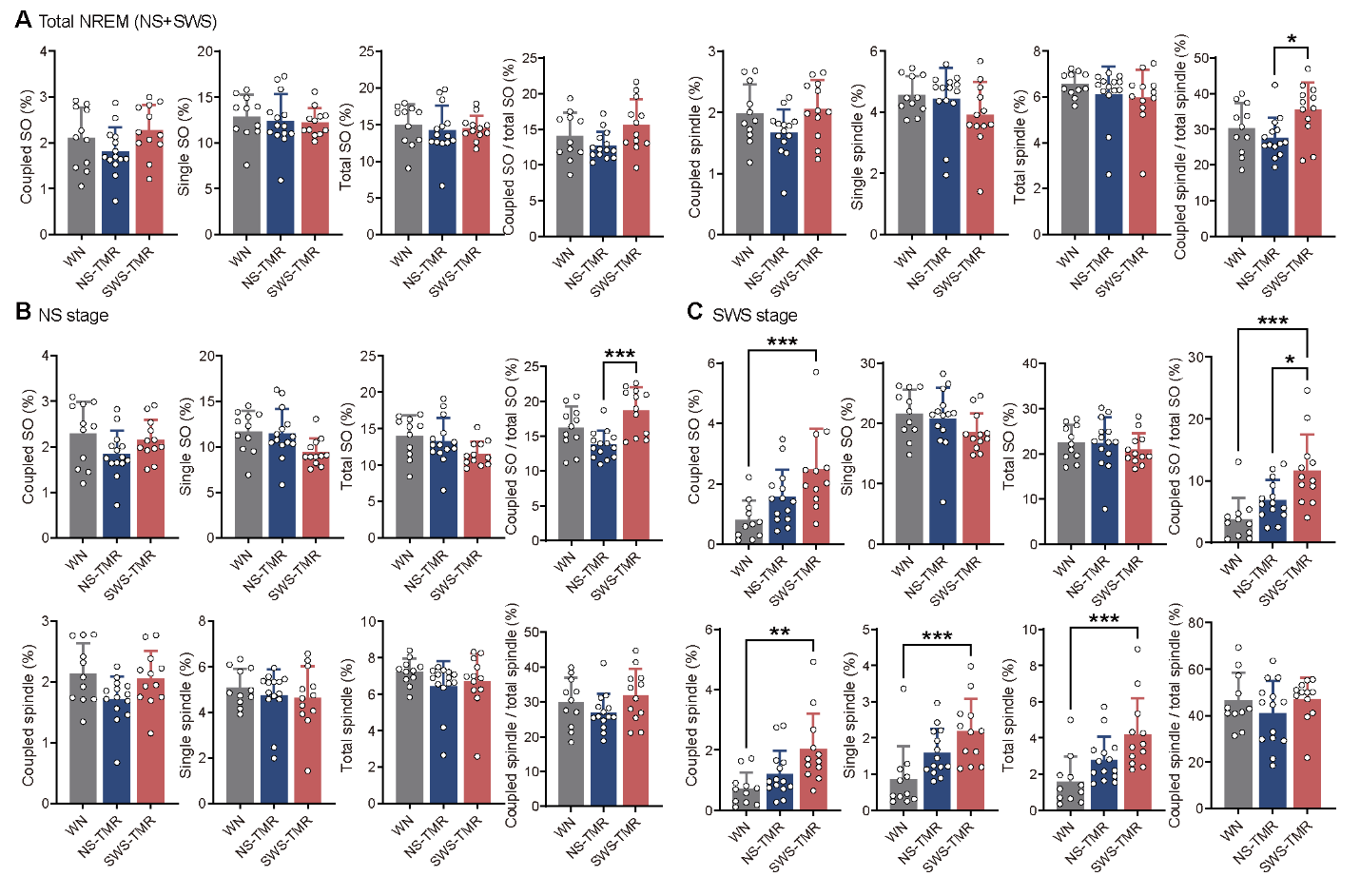


**Figure S5.**

The proportions of the time of coupled SO over total SO, coupled spindle over total spindle and quantifications of time of oscillation subtypes over the time of total NREM (**A**), total NS (**B**) or total SWS (**C**). WN, *n* = 11, NS-TMR, *n* = 14, SWS-TMR, *n* = 12; **P* < 0.05, ***P* < 0.01, ****P* < 0.001; data sets with normality and homogeneity of variance use one-way ANOVA, Tukey's multiple comparisons *post hoc* test; with normality and without homogeneity of variance use Welch's ANOVA, Dunnett's T3 multiple comparisons *post hoc* test; without normality use Kruskal-Wallis test, Dunn's multiple comparisons *post hoc* test. Data shown as mean ± SD.


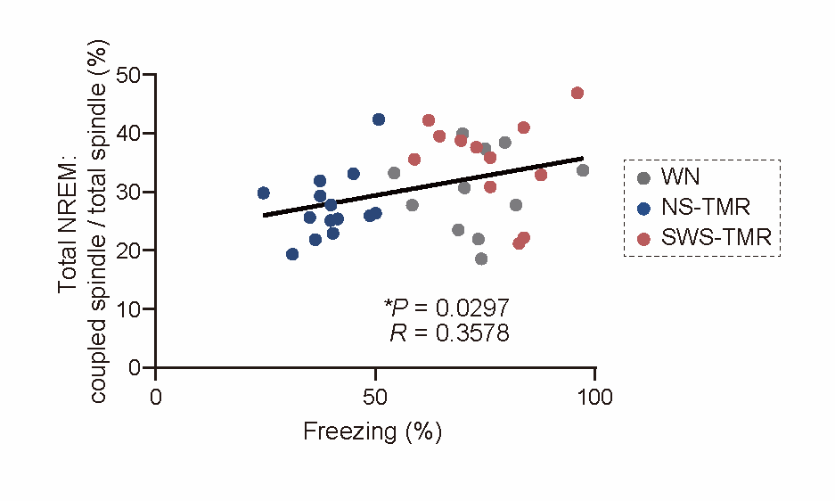


**Figure S6.**

Correlation between ratio of coupled spindle/total spindle and freezing rate in recall test at Day 3. WN, *n* = 11; NS-TMR, *n* = 14; SWS-TMR, *n* = 12; linear regression was used to plot the line and two-tailed Pearson’s correlation test was used for analysis.


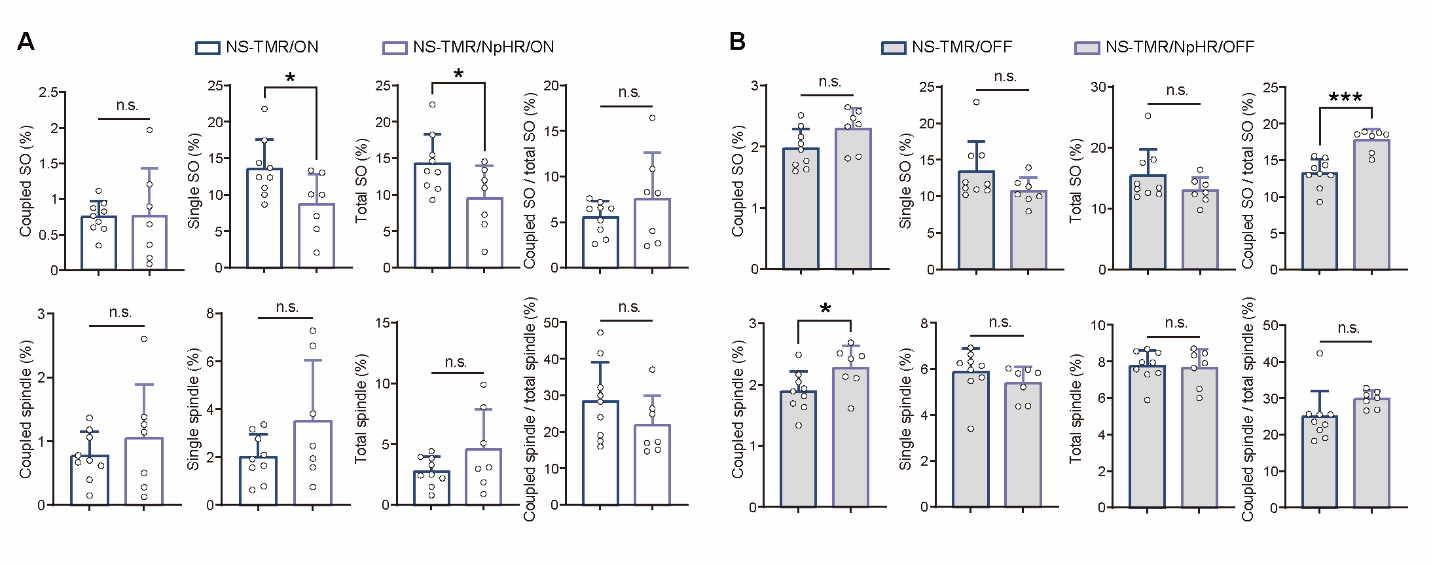


**Figure S7.**

The proportions of the time of coupled SO over total SO, coupled spindle over total spindle and quantifications of time of oscillation subtype over TMR/ON period (**A**) or TMR/OFF period (**B**). NS-TMR/ON or NS-TMR/OFF, *n* = 9, NS-TMR/NpHR/ON or NS-TMR/NpHR/OFF, *n* = 7; **P* < 0.05, ****P* < 0.001, n.s., not significant; data sets with normality and homogeneity of variance use two-tailed unpaired *t*-test; with normality and without homogeneity of variance use unpaired *t*-test with Welch's correction. Data shown as mean ± SD.


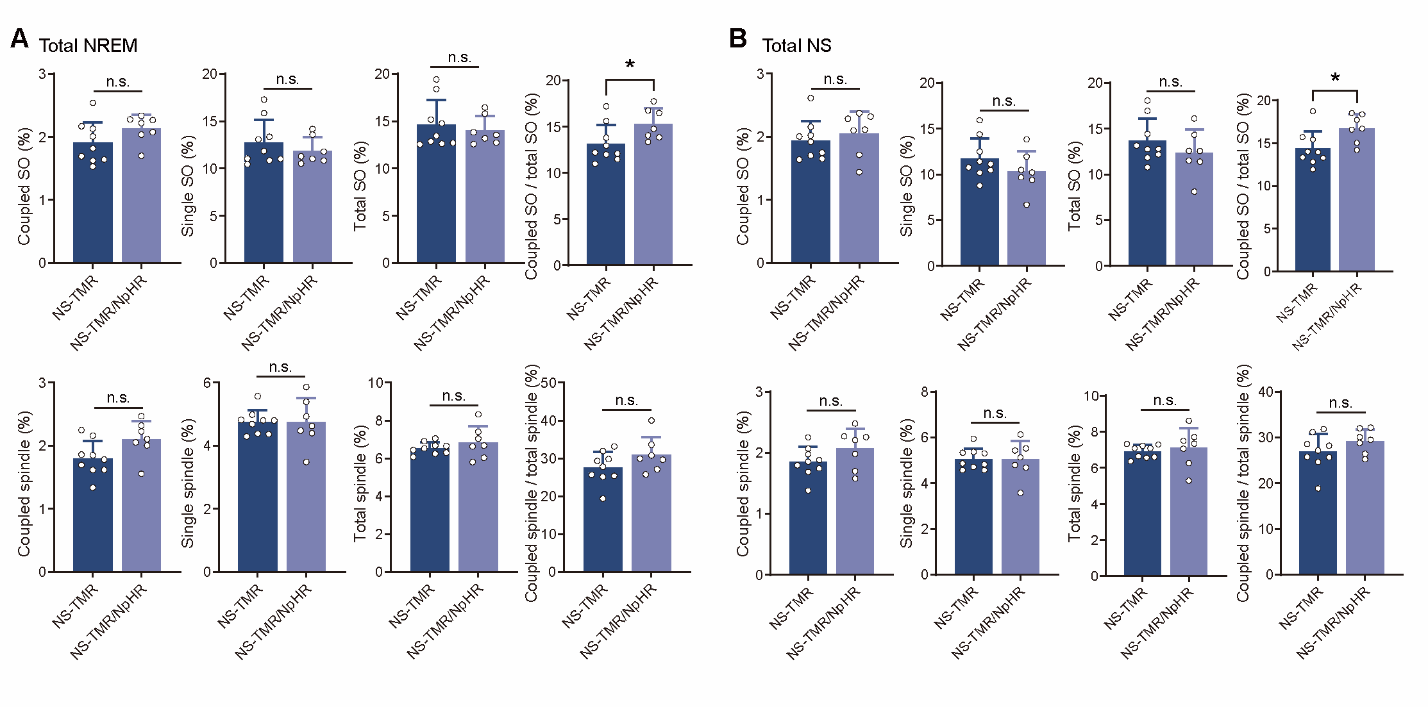


**Figure S8.**

The proportions of the time of coupled SO over total SO, coupled spindle over total spindle and quantifications of time of oscillation subtype over total NREM or NS. NS-TMR, *n* = 9, NS-TMR/NpHR, *n* = 7; **P* < 0.05; data sets with normality and homogeneity of variance use two-tailed unpaired *t*-test; with normality and without homogeneity of variance use unpaired *t*-test with Welch's correction; without normality use Mann Whitney test. Data shown as mean ± SD.


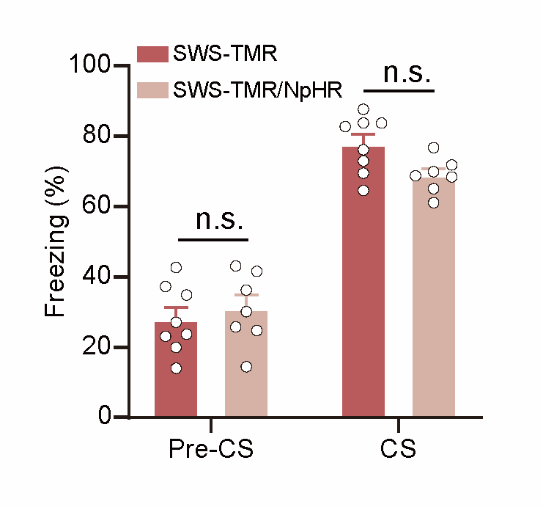


**Figure S9.**

Freezing response in recall test during the pre-CS and CS at Day 3. SWS-TMR, *n* = 8, SWS-TMR/NpHR, *n* = 7; n.s., not significant; two-tailed unpaired *t*-test. Data shown as mean ± SD.


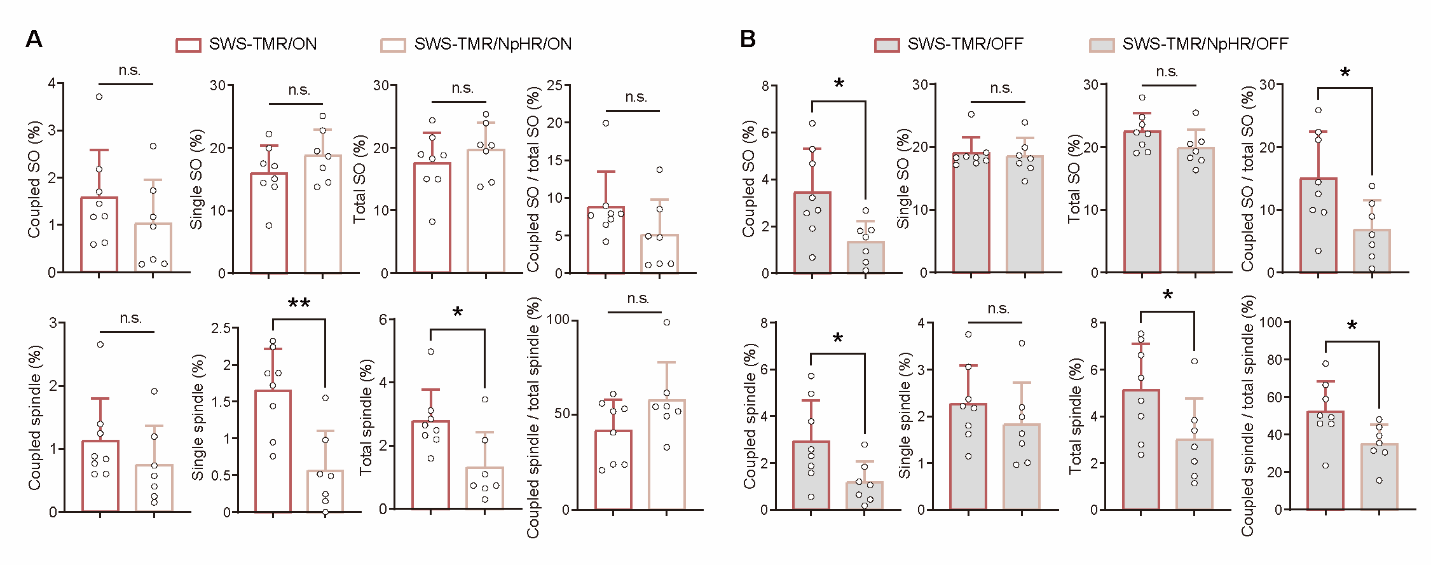


**Figure S10.**

The proportions of the time of coupled SO over total SO, coupled spindle over total spindle and quantifications of time of oscillation subtype over TMR/ON period (**A**) or TMR/OFF period (**B**). SWS-TMR/ON or SWS-TMR/OFF, *n* = 8, SWS-TMR/NpHR/ON or SWS-TMR/NpHR/OFF, *n* = 7; **P* < 0.05, ***P* < 0.01, n.s., not significant; data sets with normality and homogeneity of variance use two-tailed unpaired *t*-test; without normality use Mann Whitney test. Data shown as mean ± SD.


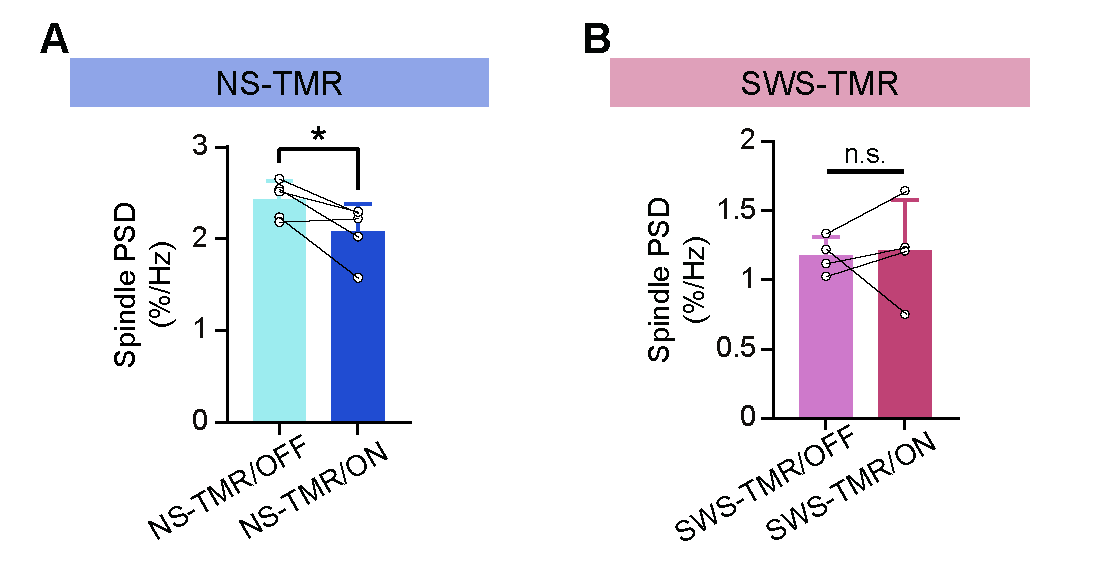


**Figure S11.**

Quantification of the PSD in spindle band (10 - 14 Hz) during TMR/OFF period and TMR/ON period. Data shown as mean ± SD. NS-TMR, *n* = 5, SWS-TMR, *n* = 4. ** *P* < 0.01, n.s., not significant; two-tailed paired *t*-test.


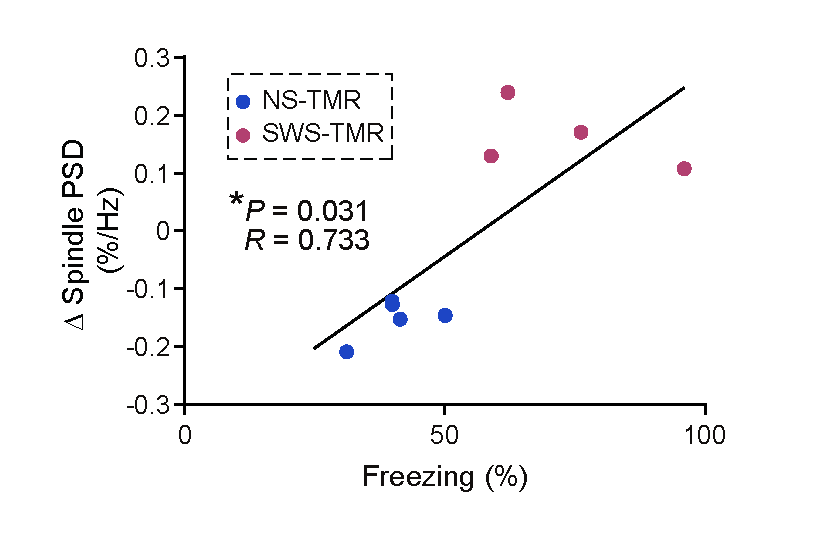


**Figure S12.**

Correlation between freezing rate at Day 3 and the change of spindle PSD between pre-TMR (5 s) and TMR (first 5 s). NS-TMR, *n* = 5; SWS-TMR, *n* = 4. Two-tailed Spearman correlation test.


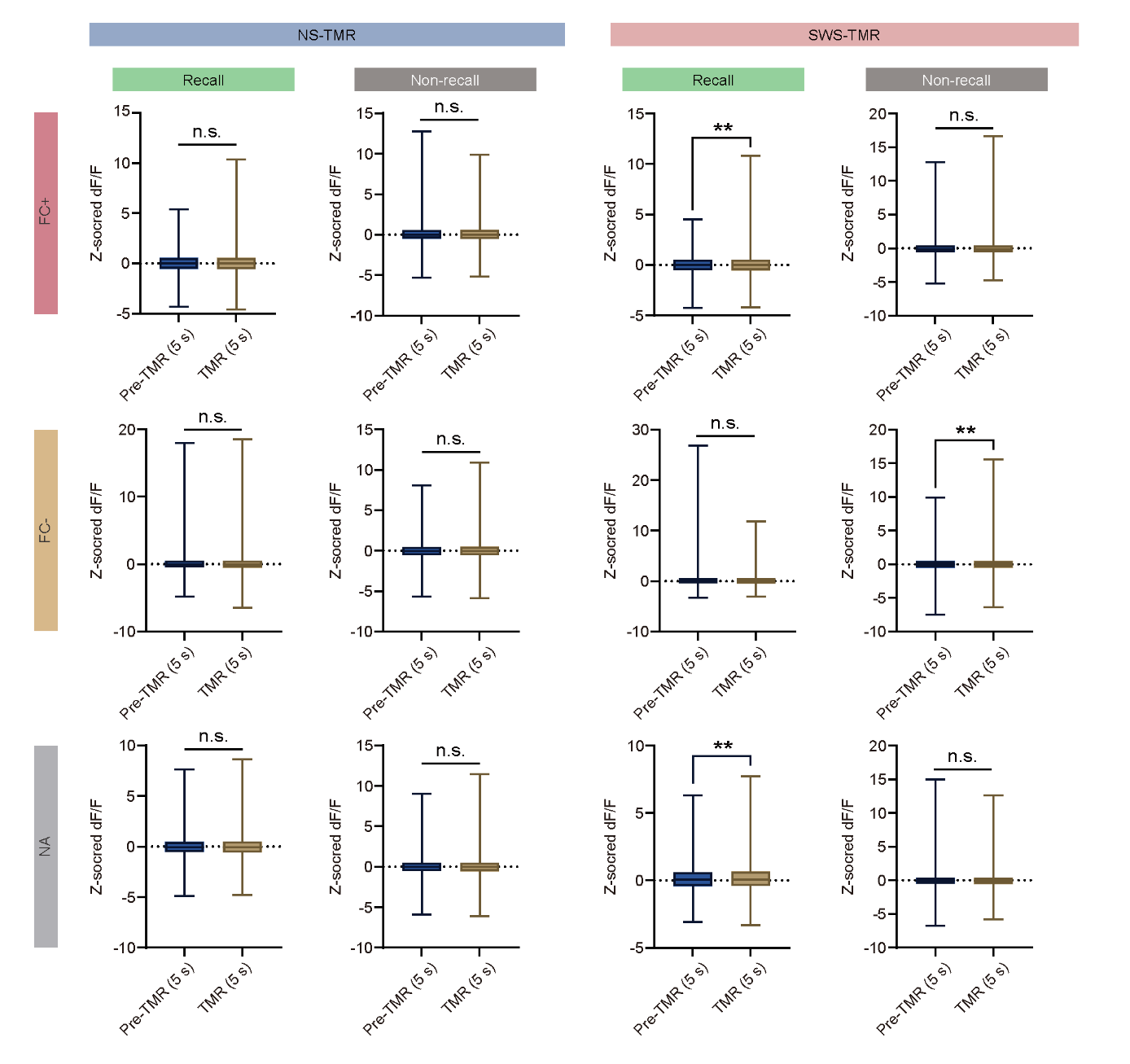


**Figure S13.**

Quantifications of z-scored dF/F of neurons during pre-TMR (5 s) and TMR (first 5 s). Data shown as box-whisker plots with box limits representing the ﬁrst and third quartile and whiskers indicating the data range. ***P* < 0.01. NS-TMR: Mice = 5, TMR trials = 235, FC+_recall_ neurons = 151, FC+_non-recall_ neurons = 512, FC-_recall_ neurons = 305, FC-_non-recall_ neurons = 800, NA_recall_ neurons = 268, NA_non-recall_ neurons = 608; SWS-TMR: Mice = 4, TMR trials = 144, FC+_recall_ neurons = 137, FC+_non-recall_ neurons = 566, FC-_recall_ neurons = 274, FC-_non-recall_ neurons = 854, NA_recall_ neurons = 259, NA_non-recall_ neurons = 670, mixed-effects model. The fixed effects and random effects for each model are listed in Table S1.


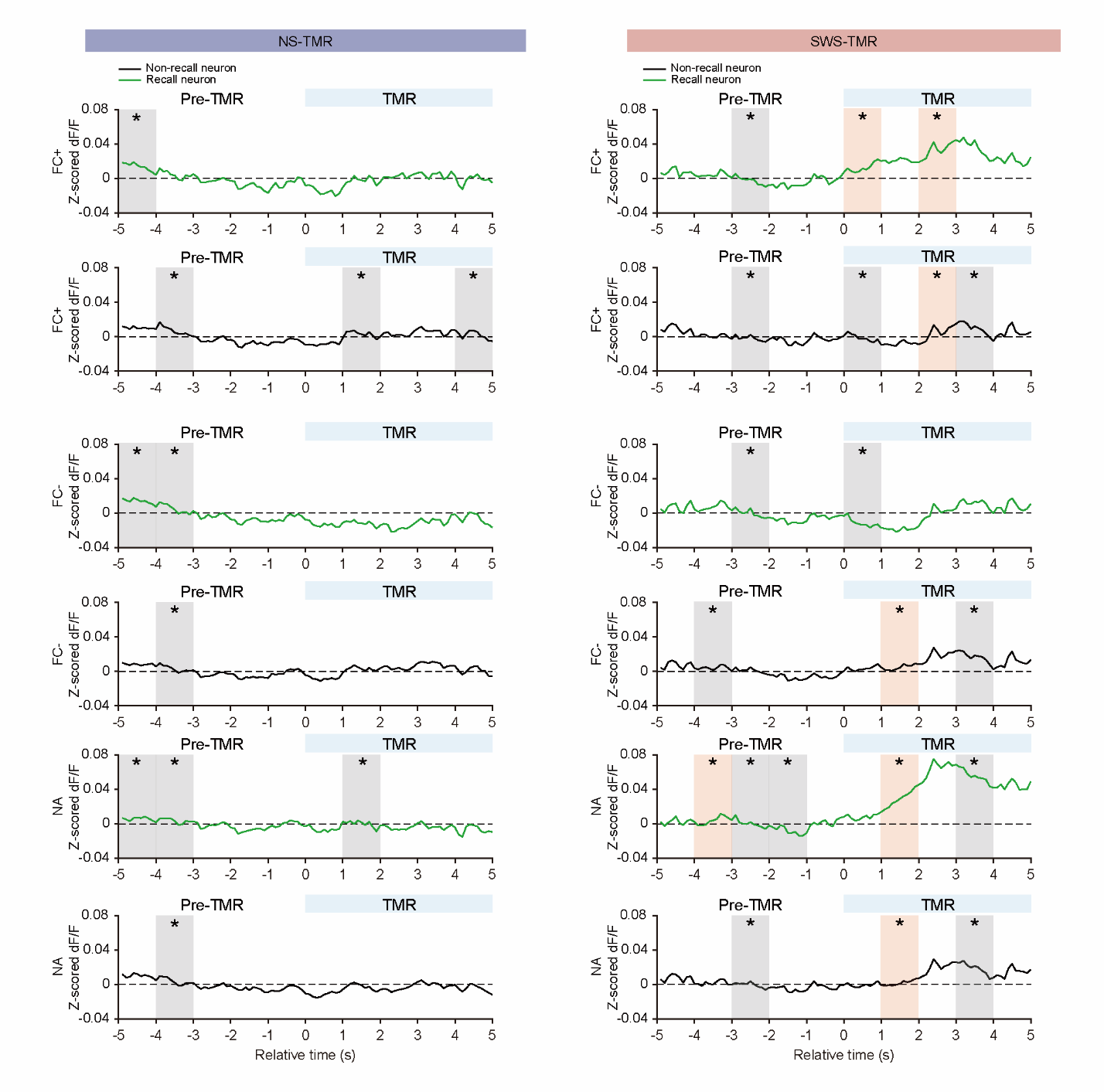


**Figure S14.**

Trend analysis of neuronal activities per second. The solid lines denote the mean z-scored dF/F. Gray shadings with asterisk show the decreasing trend and orange shadings with asterisk show the increasing trend of the second. In each second, *n* = 10 time points; two-tailed Mann Kendall Trend test; **P* < 0.05.


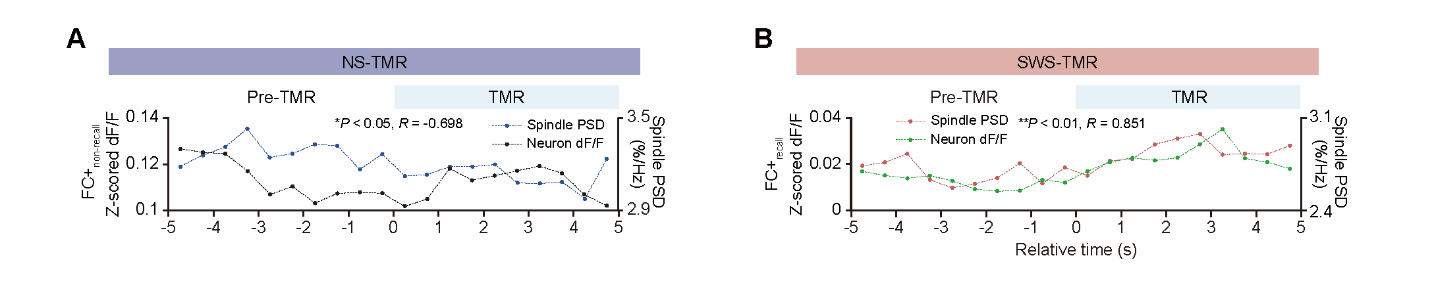


**Figure S15.**

Correlation between the mean spindle PSD and mean z-scored dF/F in each 0.5 s. (A) NS-TMR: Mice = 5, TMR trials = 235, time points (each 0.5 s) = 20; (B) SWS-TMR: Mice = 4, TMR trials = 144, time points (each 0.5 s) = 20, mixed-effects model. The correlation analyses of the rest neuron subpopulations are summarized in Table S1. The fixed effects and random effects for each model are listed in Table S1.

**
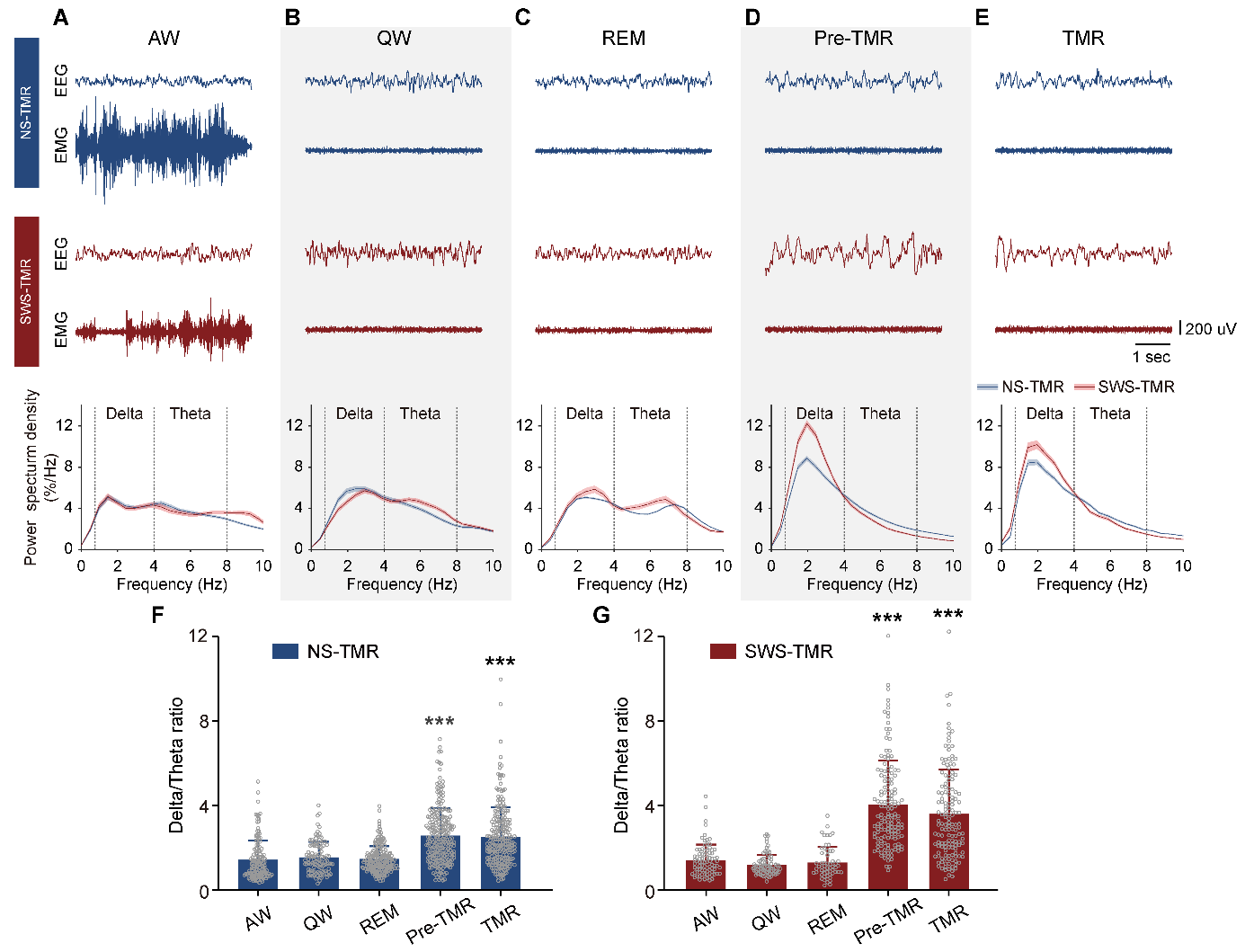
**

**Figure S16.**

(**A**) to (**E**): Upper: representative EEG and EMG trace in different vigilance states (AW: active wakefulness, QW: quiet wakefulness, REM sleep, and NS or SWS in 5 sec Pre-TMR, or during TMR) in NS-TMR (blue) and SWS-TMR (red) groups. Bottom: power spectrum density of all EEG epochs in each brain state and TMR. Delta band: 0.8 - 4 Hz, theta band: 4 – 8 Hz. Data shown as mean ± SEM. AW: NS-TMR, n = 145, SWS-TMR, n = 84; QW: NS-TMR, n = 106, SWS-TMR: 80; REM: NS-TMR, n = 253, SWS-TMR, n = 57; Pre-TMR, NS-TMR, n = 235, SWS-TMR, n = 144; TMR: NS-TMR, n = 235, SWS-TMR, n = 144. (**F**) and (**G**): Quantifications of Delta/Theta ratio in each brain state in NS-TMR and SWS-TMR groups. Delta/Theta ratio = mean PSD in delta band / mean PSD in theta band. The power spectrum analysis and Delta/Theta ratio confirmed that NS-TMR and SWS-TMR were presented in NREM sleep but not QW. Data shown as mean ± SD. ****P* < 0.001, marked groups compared to AW group; Kruskal-Wallis test with Dunn's multiple comparisons *post hoc* test.

**Table S1.**

Information of statistics in all figures. The table is in 8 columns to summarize the comparison groups with group size, homogeneity of variance test, normality test, main test, whether with post-host test or mixed effects, *P* value and other notes.
